# The integrative multi-omics approach identifies the novel competing endogenous RNA (ceRNA) network in colorectal cancer

**DOI:** 10.1038/s41598-023-46620-z

**Published:** 2023-11-09

**Authors:** Ghanbar Mahmoodi Chalbatani, Elahe Gharagouzloo, Mohammad Amin Malekraeisi, Paniz Azizi, Amirabbas Ebrahimi, Michael R. Hamblin, Habibollah Mahmoodzadeh, Eyad Elkord, Seyed Rohollah Miri, Mohammad Hossein Sanati, Bahman Panahi

**Affiliations:** 1 Cancer Research Center, Cancer Institute of Iran, Tehran University of Medical Science, Tehran, Iran; 2 Division of Cellular and Molecular Biology, Department of Biology, Nour Danesh Institute of Higher Education, Meymeh, Isfahan, Iran; 3 Student Research Committee, School of Medicine, Iran University of Medical Sciences, Tehran, Iran; 4 Psychological and Brain Science Departments, Program in Neuroscience, Indiana University, Bloomington, IN USA; 5 Wellman Center for Photomedicine, Massachusetts General Hospital, Harvard Medical School, 40 Blossom Street, Boston, MA 02114 USA; 6 Department of Applied Biology, College of Science, University of Sharjah, Sharjah, United Arab Emirates; 7 Biomedical Research Center, School of Science, Engineering and Environment, University of Salford, Manchester, M5 4WT UK; 8 Medical Genetics Department, National Institute of Genetic Engineering and Biotechnology (NIGEB), Tehran, Iran; 9 Department of Genomics, Branch for Northwest and West Region, Agricultural Biotechnology Research Institute of Iran (ABRII), Agricultural Research, Education and Extension Organization (AREEO), Tabriz, Iran

**Keywords:** Genome informatics, Computational biology and bioinformatics

## Abstract

Circular RNAs (circRNA) are known to function as competing endogenous RNA (ceRNA) in various cancers by regulating microRNAs (miRNA). However, in colorectal cancer (CRC), the precise pathological role of circ000240/miRNA/mRNA remains indeterminate. The expression level of hsa_circ_000240 was evaluated using qRT-PCR in matching pairs of CRC tumor and adjacent normal tissue samples in our laboratory. Then, to determine whether hsa_circ_000240 acted as a ceRNA in CRC, the linked miRNAs and gene targets were retrieved. Topological analysis of candidate genes using a network approach identified the most critical hub genes and subnetworks related to CRC disease. Microarray and bulk RNA sequencing analyses were utilized to comprehensively evaluate the expression levels of both miRNA and mRNA in CRC. Single-cell RNA-seq analysis was also used to evaluate the significant overall survival (OS) genes at the cellular level. ATAC-seq data provided insights into candidate genes' accessible chromatin regions. The research uncovered a considerable upregulation of hsa_circ_000240 in CRC tissues. Three miRNAs interacted with the target circRNA. One thousand six hundred eighty intersected genes regulated by three miRNAs were further identified, and the relevant functionality of identified neighbor genes highlighted their relevance to cancer. The topological analysis of the constructed network has identified 33 hub genes with notably high expression in CRC. Among these genes, eight, including CHEK1, CDC6, FANCI, GINS2, MAD2L1, ORC1, RACGAP1, and SMC4, have demonstrated a significant impact on overall survival. The utilization of single-cell RNA sequencing unequivocally corroborated the augmented expression levels of CDC6 and ORC1 in individuals with CRC, alongside their noteworthy connection with the infiltration of immune cells. ATAC-seq analyses revealed altered accessibility regions in Chr2, 4, and 12 for CDC6 and ORC1 high-expression. Correlation analysis of CDC6 and ORC1 further highlighted the association of candidate gene expression with exhaustion markers such as CTLA4, CD247, TIGIT, and CD244. The candidate genes exhibit a positive correlation with chromatin remodeling and histone acetylation. These epigenetic modifications play a significant role in influencing the cancer progression following expression of CDC6 and ORC1 in CRC. Additionally, results showed that the methylation rate of the promoter region of CDC6 was elevated in CRC disease, confirming the functional importance of CDC6 and their interaction with hsa_circ_000240 and associated ceRNA in CRC. In conclusion, this study highlights hsa_circ_000240's role as a ceRNA in CRC. It opens new avenues for further dissection of CDC6, ORC1, and underlying novel epigenetics and immunotherapy targets for CRC therapy.

## Introduction

Colorectal cancer (CRC) is the fourth most prevalent neoplasm worldwide, making it a prominent health concern. Furthermore, it is the second most common cause of cancer-related mortality, with an estimated 881,000 deaths in 2018^1^.

Compared with other malignancies, there are insufficient diagnostic biomarkers for CRC. Thus early diagnosis is uncommon. Furthermore, CRC has a poor cure rate with conventional cancer treatment, such as surgery, chemotherapy, and radiotherapy, with only 10–20% of patients surviving for 5 years^1^. Recently immunotherapy has been tested for CRC treatment based on immune checkpoint inhibitors such as anti-PDL1 antibodies (atezolizumab, durvalumab, and nivolumab). Additionally, the next revolution in cancer management could be achieved by combining progress in novel targeted therapies with advancements in cancer immunotherapy^2^. To this end, it is necessary to thoroughly investigate the mechanisms involved in developing CRC to discover new anti-cancer therapeutic targets.

Circular RNAs (circRNAs) were first discovered in 1976 but have recently been introduced into oncology as valuable cancer biomarkers. With the help of advances in high-throughput sequencing and bioinformatics tools, a wide range of circRNAs have been discovered to be expressed in tumor cells and tissues and involved in many human diseases^3,4^. With a covalent closed-loop structure devoid of a poly-A tail, circular RNAs represent an innovative category of non-coding RNAs^5^. These properties make them more stable and conserved than linear RNAs. They are more abundant because they are not easily destroyed, making them highly sought after as cancer biomarkers. Indeed, the activity of circRNAs can be categorized as (a) circRNAs can function as competing endogenous RNA (ceRNA) molecules to act as a miRNA sponge to regulate gene expression by inhibiting the effect of the miRNAs on their target mRNAs, (b) circRNAs can directly affect gene expression and modulate the stability of mRNAs through interaction with RNA binding proteins; (c) in some tumor cell lines, circRNAs can also serve as protein frameworks to generate functional proteins^6–8^. Nowadays, it has been realized that circRNAs may function as biomarkers and can also be involved in cancer promotion, invasion, and metastasis in CRC, lung cancer, and bladder cancer^9^.

Bulk RNA sequencing assesses the average gene expression of diverse bulk cell populations. In this approach, the total RNA of multiple cell types will be extracted and pooled together. These results illustrate the average expression profile of cells, providing valuable information to discern gene expression variations between different tissues. Nonetheless, it becomes challenging to differentiate individual cells within the population, leading to the potential obscuring of rare cell populations or subtle transcriptional differences. Indeed, a revolutionary development in genomic research, single-cell RNA sequencing (scRNA-seq), has made remarkable advancements in cancer studies.

The most valuable aspect of scRNA-seq is its ability to examine transcriptional expression at the cellular level. This differentiates it from bulk techniques that assess average gene expression in a mass of samples^10^. In this contemporary investigation, we have validated the findings through diverse omics methodologies, including microarray, bulk RNA sequencing, single-cell RNA sequencing, and ATAC-seq.

This contemporary investigation aimed to ascertain the function of hsa_circ_00240 (circID: Hsa_circ_0000810) extracted from the circ2Trait and CircBase databases. According to CircBase, hsa_circ_000240 is positioned at chr17:76,967,343–76,968,212, associated with the LGALS3BP gene, and significantly correlated with CRC (*p*-value < 0.05).

In our laboratory, we conducted qRT-PCR to assess the expression level of hsa_circ_000240 in the corresponding CRC tumor and neighboring tissue samples. Then, to determine whether hsa_circ_000240 acted as a ceRNA in CRC, the linked miRNAs and gene targets were retrieved, and the hsa_circ_000240 network was created. Additionally, an intricate network of interactions between proteins was constructed, allowing for the identification of a set of hub genes. The gene ontology and pathway analysis were conducted to unveil the potential pathogenic role of circ000240 in CRC. The mRNA expression levels of essential genes were obtained from CRC patients with colon and rectal cancer in TCGA. The overall survival rates of hub genes were retrieved from over 600 CRC patients. To evaluate the expression of hub genes at the cellular level, scRNA-seq analysis was performed, and the accessibility of chromatin regions of confirmed hub genes was identified through ATAC-seq data derived from the TCGA database. Furthermore, the correlation between immune cell infiltration and hub genes was comprehensively evaluated (Table 1).

**Table 1:**
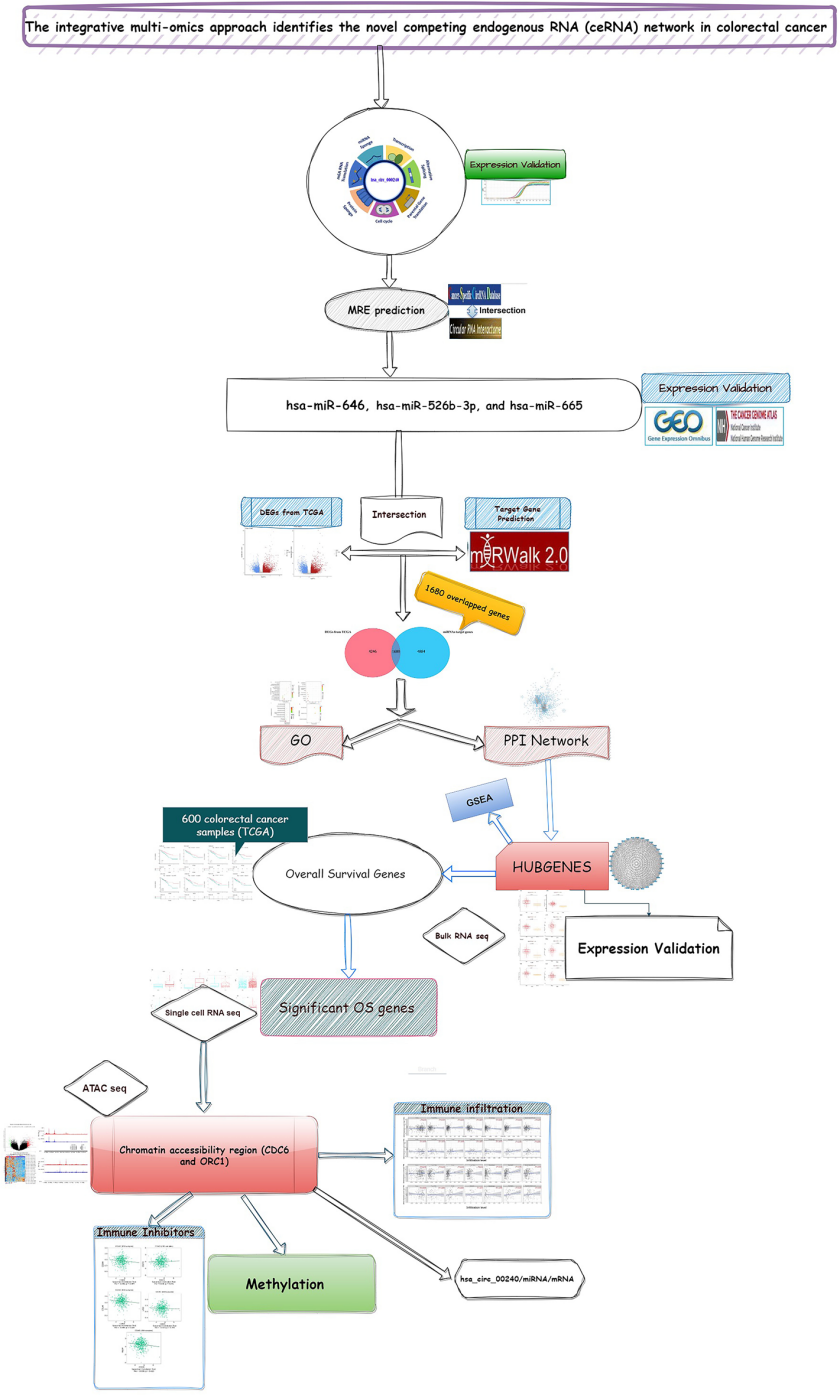
Flowchart of the current study depicting CRC research elements, including data sources like GEO and TCGA. The study involves analysis of MRE (miRNA response element) interactions, DEGs, and PPI networks using MCODE (Molecular Complex Detection). Additionally, GO analysis will be performed for functional annotations. The study incorporates diverse techniques, such as microarray, Bulk RNA seq, scRNA seq, ATAC seq, and methylation analysis.

## Materials and methods

### Ethical approval

The Institutional Review Board and Ethics Committee of Tehran University of Medical Science, Cancer Institute approved this study, and all methods were performed by the Declaration of Helsinki. By ethical approval with complete institutional address Iran, Tehran University of Medical Science, with number 1394.2197 (IRC.TUMS.REC.1394.2197), approved the study, and all patients signed informed consent.

### Biological tissues

A comprehensive collection of 31 matched fresh frozen tumors and adjacent standard tissue samples were acquired from colorectal cancer patients through the Iran Tumor Bank. After surgical resection, tissue samples were promptly immersed in RNA-fix reagent and kept at − 80 °C until utilization. The information on the stage of the tumor has been compiled in the supplementary Excel 1.

### Isolation of RNA and performing qRT-PCR

Ttotal RNA extraction was accomplished using TRIzol reagent (QIAGEN, USA) following the protocol provided by the manufacturer. A reverse transcription reaction was conducted using a TaKaRa kit from 5 μg of total RNA, as provided protocol by the company. The qRT-PCR was duplicated using the TaKaRa SYBR GREEN Master Mix on the Bio-rad Real-Time PCR Device. The primers were synthesized by SINAGENE (Tehran, Iran). The primer sequences used for hsa_circ_000240 were as follows: 5′-TTGCTCCTGGCGCTATAC-3′ (forward) and 5′-AGAGTCCAGCGGCAAACTA-3′ (reverse). For the control housekeeping gene glyceraldehyde-3-phosphate dehydrogenase (GAPDH), the primers used were 5′-TCGACAGTCAGCCGCATCTTTT-3′ (forward) and 5′-ACCAAATCCGTCGACCTCTT-3′ (reverse). The ΔCt method was used to evaluate the data, and findings were expressed as mean ± SD.

### Prediction of microRNA response elements

DIANA-miRPath v3.0 is an online website dedicated to determining miRNAs' regulatory functions and predicting their associated regulatory pathways (Fig. 4). We used information from the circ interactome and Cancer-Specific circRNA (CSCD) databases to predict miRNA targets for the microRNA response elements (MREs) of hsa_circ_000240 (Table 2). After that, the miRNAs that overlapped were chosen for further mRNA prediction.

### Proliferation role of miRNAs in CRC

To gain insight into how miRNAs influence proliferation, we utilized Venny to analyze 1680 overlapped genes. We retrieved proliferation-related genes from the Gene Ontology resource webserver (https://www.geneontology.org/) to identify interacting proliferation genes in CRC.

### Expression of microRNAs verified by GEO data

GEO (gene expression omnibus) was used to obtain the miRNA expression profile. Various search terms, including "colorectal," "rectal," "cancer," "carcinoma," "neoplasm," "colon," "CRC," "tumor," and "malignant," along with "miRNA," "microRNA," "miR," "noncoding RNA," and "ncRNA," were utilized to identify relevant studies related to CRC and colon cancer, with a focus on tumors of size 3cm. The inquiries for each miRNA involved the miRNA type, location, case incidence, and expression level. Details of the papers, such as the first author’s name, year of publication, data source, etc., were retrieved.

### MRNA prediction for miRNAs

The target mRNAs were predicted using miRWalk V 3.0. Furthermore, target genes were predicted using a minimum of eight algorithms. In this study, 12 prediction algorithms (including miRMap, miRanda, miRDb, miRWalk, picta2, RNA22, airbridge, Trgetscan, RNAhybrid, PITA, miRDB, and Microt4) were employed to be sure of the accuracy of the prediction results in^11^.

### Differentially expressed genes (DEGs) and the overlapped mRNAs in CRC.

TCGA (https://www.cancer.gov/tcga.) is a web server that shows the main tumor-related genomic modifications. Using screening criteria of |log2 (fold change)|> 2 and FDR < 0.05, differentially expressed genes (DEGs) were defined with the R studio package (edgeR version 4.3) and Deseq2 (Version 3.17) package) in Bioconductor^12^. The overlapped target miRNA genes and DEGs were extracted via a Venn plot.

### Gene ontology (GO) enrichment analysis of overlapping genes

DAVID is a freely available data server online tool that provides a functional analysis of an extensive list of genes/proteins. It elucidates the pathways, cellular biology, and gene functions. The enrichment evaluation of the overlaps of the genes was set at *P *value < 0.05 and the number > 2.

### PPI-network and hub genes

The STRING web server provides credible protein interaction data and comprehensive annotations. In the milieu of this investigation, protein interactions were regarded as statistically significant when their combined score surpassed 0.7 (Version 11 STRING, PPI-Network). Nangraj^13^ networks were constructed using the genes that overlapped in the dataset. Incorporating molecular interaction network data was accomplished through the utilization of Cytoscape, a freely available open-source software platform. This highly versatile molecular and biological research tool demonstrates the capability to load diverse biomolecular interaction information, harmonize extensive datasets, and construct visually informative mappings, facilitating comprehensive analyses and functional annotations across these datasets. The functionality of Cytoscape can be expanded with various Apps. MCODE, a prominent algorithm, utilizes node-weighting arithmetic to identify densely interconnected regions within extensive molecular interaction networks, specifically in PPI networks. As a result, Cytoscape version 3.7.1 was employed to visualize the PPI networks. Essential modules containing hub genes and other significant modules within the networks were identified using MCODE, with specific criteria: a degree cut-off of 2, a node score cut-off of 0.2, a maximum depth of 100, and a k-score of 2.

### Validation of hub genes and enrichment analysis

We procured transcriptome and patient information from The Cancer Genome Atlas (TCGA) for colon and rectal cancer, comprising 647 CRC samples and 51 normal controls. To filter and normalize TCGA-retrieved data, we employed the edgeR and limma packages in Bioconductor. The expression profiles of the 33 hub genes were validated using TCGA CRC samples. To delve into the effect of potential hub genes on overall survival (OS), the interplay between OS and hub genes was tested using clinical data of patients. Survival and survminer packages were utilized to perform survival analysis and data visualization. In this study, the significance of the hub genes was assessed using the Kaplan–Meier method and log-rank test (set at *p*value < 0.05). In the context of this research, the hazard ratio of hub genes and potential additional hub genes was calculated using Cox proportional hazards regression. The expression profiles of significant hub genes in CRC samples and normal controls were visualized using box plots. Furthermore, we conducted gene ontology enrichment on 33 hub genes to understand the precise function of central hub genes.

### Diagnostic and prognostic value evaluation

The diagnostic efficacy of hub genes associated with significant overall survival in TCGA data was assessed using receiver operating characteristic (ROC) curves. We employed the pROC package in R to obtain the area under the curve (AUC) to measure the diagnostic accuracy.

### Contribution hub-genes in known oncogenes and tumor suppressor pathways

We submitted 8 hub genes to the STRING database at [https://string-db.org/] and obtained 100 interacting genes with these hub genes in the experimental mode of active interaction sources. Then, we performed an overlap analysis of the interacting genes with the genes of important pathways in colorectal cancer, such as Wnt signaling pathway, MAPK signaling pathway, JAK-STAT signaling pathway, PI3K-Akt Pathway as oncogenic pathways, and p53 signaling pathway as a tumor suppressor pathway, using the online tool Venny 2.1 at [https://bioinfogp.cnb.csic.es/tools/venny/].

### Gene set enrichment analysis

Using computer-based knowledge, gene enrichment analysis (GSEA) investigates the biological functions of genes^12^. Correlations of selected hub genes with other genes were calculated in the count matrix to order the gene set. GSEA analysis for hub genes was performed using clusterProfiler, a package in R, to obtain biological pathways, and the GO database was used as a reference. Biological pathways related to hub genes were extracted and visualized with the Enrichplot in R.

### Data acquisition and processing for scRNA-seq

siRNA seq data with an accession number GSE144735 from the Gene Expression Omnibus^14^ were obtained to confirm the data. This data comprised 18 samples from 6 CRC patients: six representatives from the tumor core, six from the tumor border, and six from the normal mucosa. In total, 17,990 cells from the tumor core and control mucosal tissue samples were included for analysis. Seurat (version 4.1.1), an understanding of the R package, was used for quality control of the raw, unique molecule identifiable count matrix^7^. Cells with several genes < 500 or > 4000 were ruled out. The mitochondrial proportion limit was set to < 15%. In total, 15,238 cells remained after filtration. Normalize Data in the Seurat package was used to perform data normalization with the log-normalize method. Data visualization was done with ggplot2 in R.

### Differential chromatin accessibility analysis and visualization of ATAC-seq data

ATAC-seq data of 410 samples for 23 cancer types were obtained from the TCGA platform, and following data processing, 79 examples were selected to investigate chromatin accessibility in colorectal cancer^15^.

CRC samples were grouped into highly-expressed and lowly-expressed pieces for confirmed hub genes based on the median of confirmed hub genes expression, and the normalized count matrix was processed using R software to compare the accessibility of chromatin regions. Conversion to counts per million (CPM) and log2 transformation were applied to the count matrix during normalization. Chromatin regions with FDR < 0.05 and |log2 count difference|> 1 were determined as significant peaks. Normalized bigWig files associated with colorectal cancer were obtained from the TCGA database, and karyoploteR and ggplot2 packages were employed to visualize genomic region peaks.

### Role of CDC6 and ORC1 in downstream epigenetic modifications

To understand the specific epigenetic function of CDC6 and ORC1, we have used the EpiFactors database. In brief, EpiFactors is an available online database of epigenetic regulators and their expression profiles that provide required information based on the four contents: Genes, Complexes, Histones and protamines, lncRNAs, and Expression, either directly or using the keyword search. We sought to pinpoint the role of CDC6 and ORC1 in downstream epigenetic modification in more detail. To this end, genes related to chromatin remodeling and histone modification were extracted from the GO database, and two protein–protein interaction networks, one involving CDC6 and chromatin remodeling genes and the other one involving ORC1 and histone modification genes, were constructed using the STRING database. In the next step, the first-order neighbor genes for CDC6 and ORC1 were selected to apply correlation analysis using the Spearman method. *P* value < 0.05 was considered statistically significant.

### The correlation between the expression of CDC6/ORC1 and the infiltration of immune cells in tumor tissues

The accessible online webserver, tumor immune estimation resource (TIMER), enables the systematic evaluation of various immune cell types in tumor samples. The expression patterns of central genes and the presence of immune cells were assessed in CRC samples. The list of immune cells included B cells, CD8-positive T cells, neutrophils, CD4-positive T cells, macrophages, and dendrite cells.

### Prediction of response to immune chekpoint inhibitor (ICI) treatment

Response to immunotherapy in colorectal cancer samples was evaluated using the tumor immune dysfunction and exclusion (TIDE) algorithm. Tide receives a normalized gene expression profile and calculates the TIDE score based on the expression of T cell dysfunction and infiltration genes, thereby estimating the potential of immune escape in cancer samples. Lower TIDE scores suggest greater responses to immune checkpoint blockade therapy.

### ceRNA network construction for CDC6 and ORC1

The ceRNA network for circ000240 was constructed using the Cytoscape platform v3.8.2, which is freely available as an open-source tool Supplementary Fig. 5.

### Statistical evaluation

GraphPad Prism 8.0 was used for statistical evaluation (GraphPad Software, La Jolla, CA). The qRT-PCR results were analyzed through ΔCt. Hsa_circ000240 levels were analyzed using a one-way analysis of variance^16^ to assess the associations ( 2).

**Table 2:** The physical interaction between hsa_circ_000240 with miRNAs.

CircRNA Mirbase ID	CircRNA^33^—miRNA (Bottom) pairing	Site type	CircRNA Start	CircRNA end
hsa_circ_0000810 (5′ … 3′) hsa-miR-646 (3′ … 5′)	CUCCCCACCAUCCAGAGCUGCUG ||||||| CGGAGUCUCCGUCGACGAA	7mer-m8	259	265
hsa_circ_0000810 (5′ … 3′) hsa-miR-526b-3p (3′ … 5′)	CCUGAGCGCUCAGCUUCAAGAAA |||||| UGUCUUUCACGAAGGGAGUUCUC	7mer-1a	698	704
hsa_circ_0000810 (5′ … 3′) hsa-miR-665 (3′ … 5′)	UUUGUGACAGACAGUUCCUGGAG |||||| UCCCCGGAGUCGGAGGACCA	7mer-1a	61	67

## Results

### Assessment of hsa_circ_00240 expression in tumor tissue

**Figure 1:**
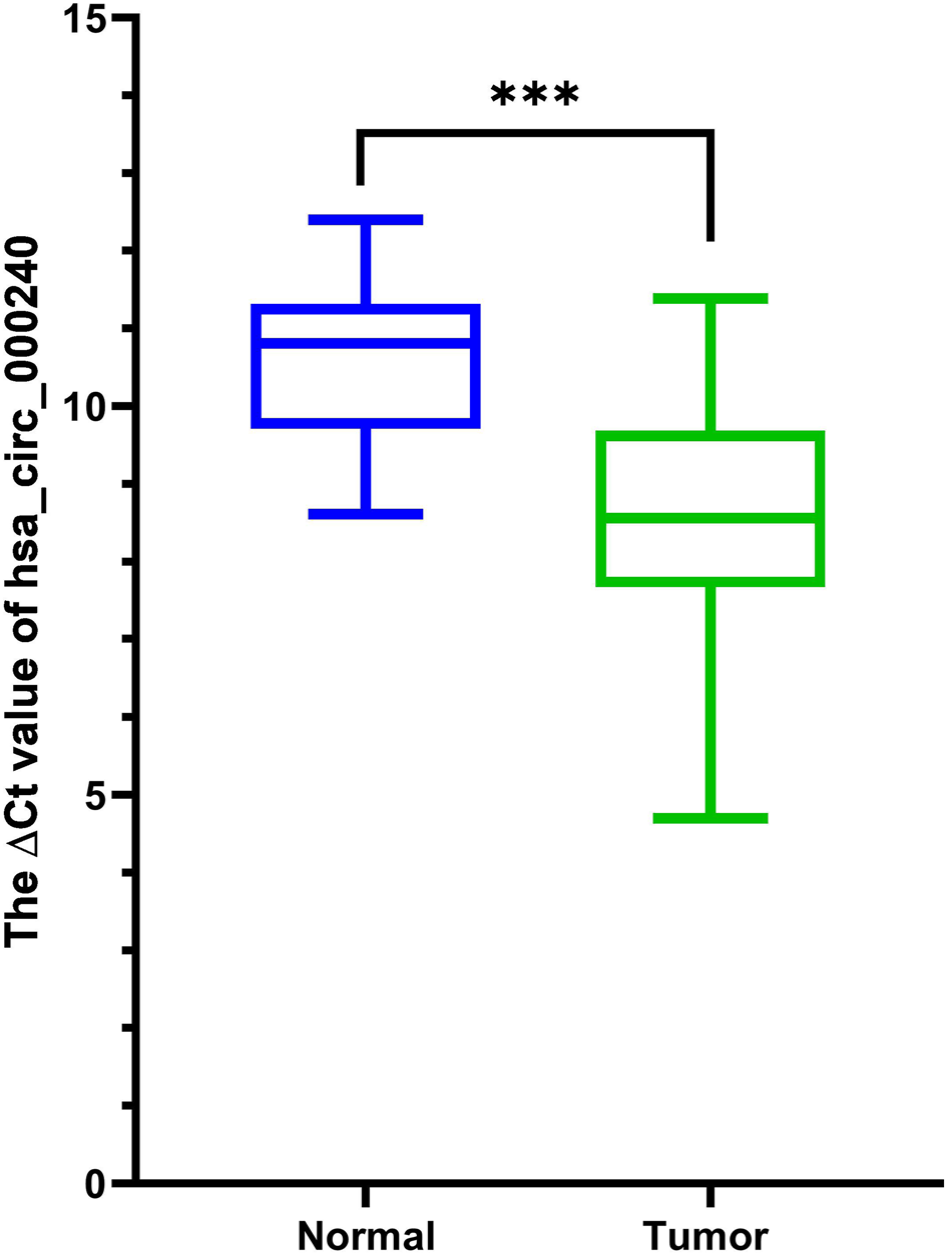
Expression analysis of hsa_circ_000240 in 22 paired tissue samples from colorectal cancer (CRC) patients.

During this investigation, a comprehensive collection of 22 paired samples, encompassing tumor tissues and their respective neighboring healthy tissues, was utilized for qRT-PCR analysis. The expression level of hsa_circ_000240 in CRC samples was significantly higher than the corresponding nearby healthy tissue (*P* < 0.05), as depicted in Fig. 1.

### Functional and biological process of hsa_circ_000240

In this study, according to the CircBase database, the hsa_circ_000240 was derived from LGALS3BP as exogenic circRNAs. To further analyze the biological function and process of the hsa_circ_000240, GO enrichment was performed on the source genes of circRNA. A total of three GO terms were enriched. In terms of cellular process, the source gene of circRNA was significantly enriched into cell adhesion, and regarding function, it has contributed to scavenger receptor activity Table 3.

**Table 3:** GO functional annotation and enrichment analysis of Lgals3BP of hsa_circ_000240.

GO	Terms
BP	Cell adhesion, cellular defense response, and signal transduction
MF	Scavenger receptor activity
CC	Blood microparticle, collagen-containing extracellular matrix, extracellular exosome, extracellular region, extracellular space, membrane, and platelet dense granule lumen

### miRNA expression validation in CRC through microarray

**Figure 2:**
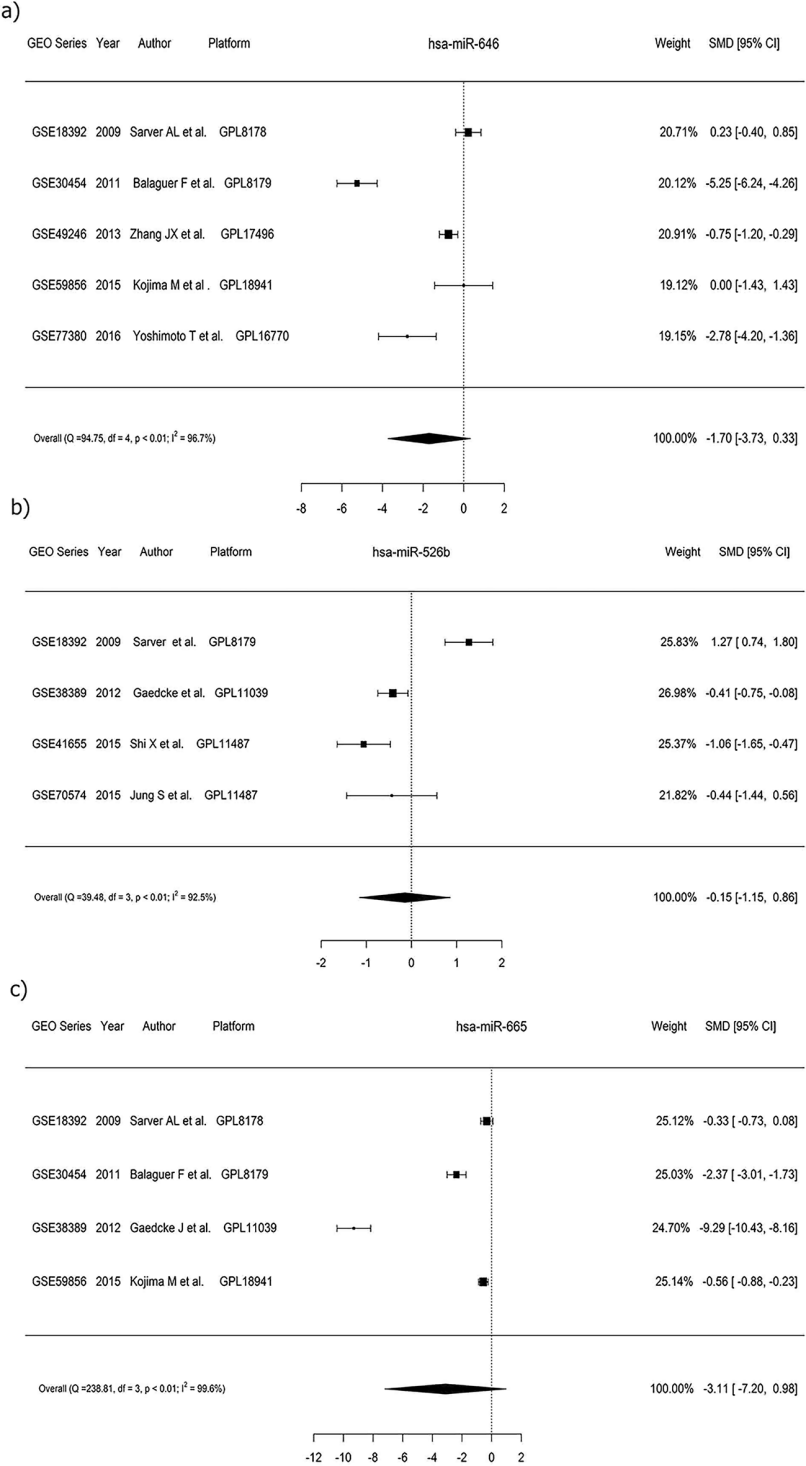
Forest plot depicting the expression levels of three miRNAs in CRC GEO datasets: **a** miR-646, **b** miR-526b-3p, and **c** miR-665. The plot showcases the expression data and provides insights into the differential expression patterns of these miRNAs in CRC samples.

**Figure 3:**
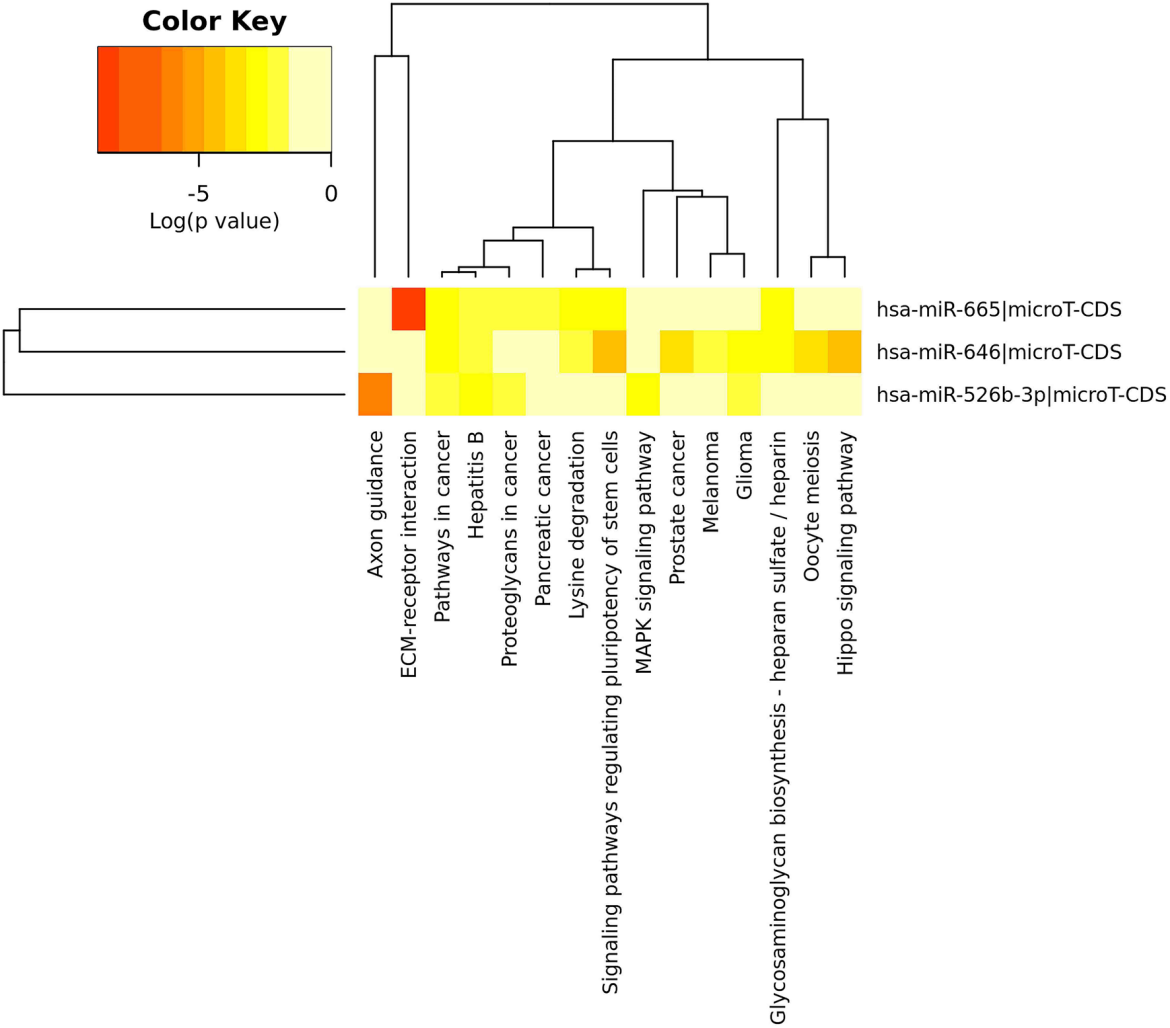
The heatmap presents the significant signaling pathways regulated by the selected miRNAs, and it was generated using DIANA-miRPath.

In data sets extracted from GEO, the results of this evaluation showed low expression of miR-646 (SMD = − 1.70, 95% CI − 3.73 to 0.33, *p* < 0.01), miR-526b-3p (SMD =− 0.15, 95% CI − 1.15 to 0.86) and miR-665 (SMD = − 3.11, 95% CI − 7.20 to − 0.98,) in CRC tissues (Fig. 2a–c). The significance level was set at *p* < 0.01. As shown in Fig. 3, these microRNAs take part in various signaling pathways such as pathways in cancer, proteoglycan in cancer, hippo signaling pathways, etc.

### Identification of hub genes and network construction

**Figure 4:**
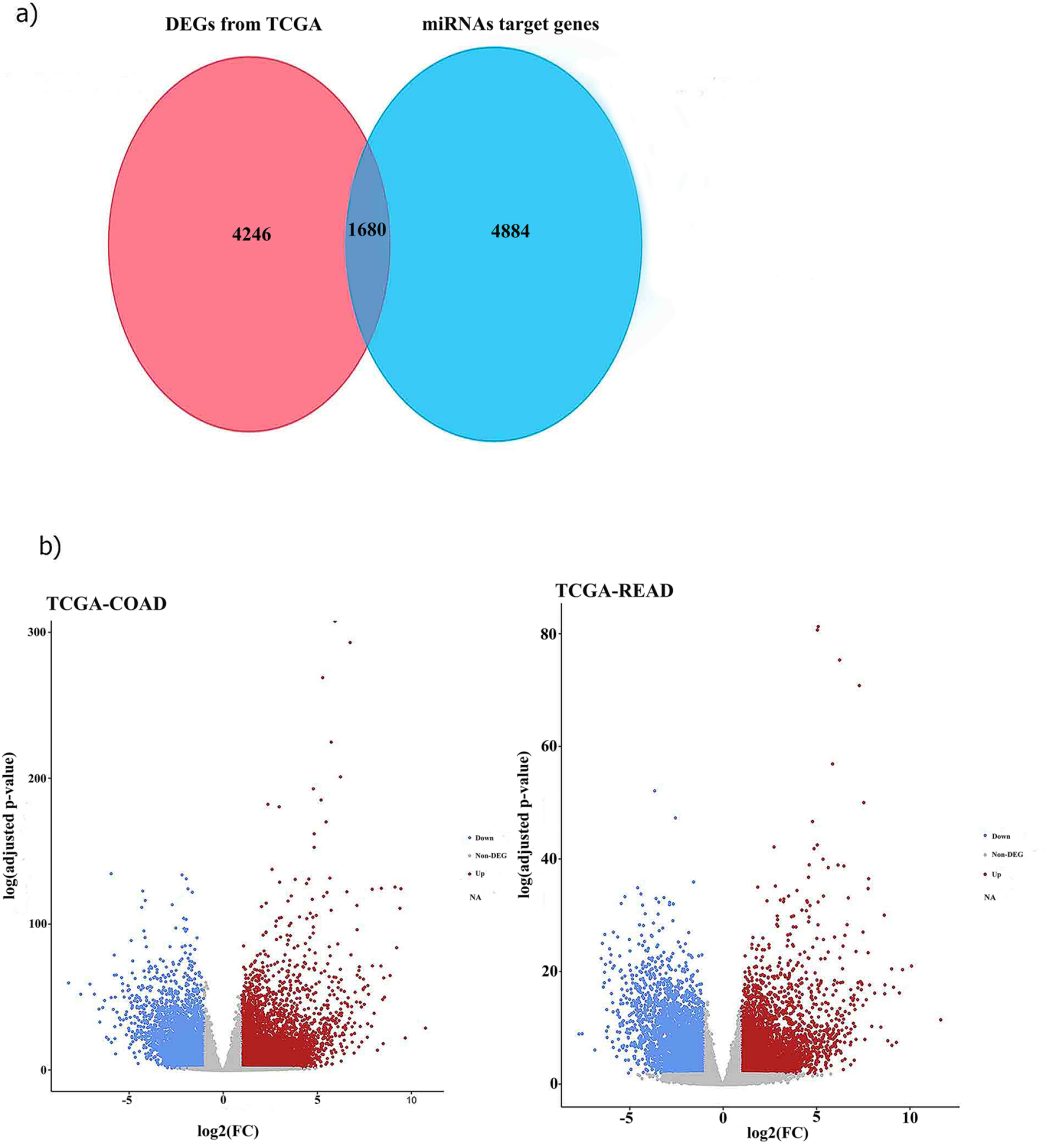
Depicts the discovery of 1680 genes of paramount significance in colorectal cancer (CRC). The figure is composed of two segments: **a** A global plot that depicts the concurrence between genes exhibiting DEGs and the genes targeted by microRNAs. **b** A Volcano plot presenting the DEGs in CRC based on TCGA data. The plot, generated through the utilization of the R package 'ggplot2', provides a visual representation of the distinct expression patterns of genes in CRC compared to normal samples.

1680 genes overlapped in the combination of the two databases CRC TCGA and miRWALK (Fig. 4a). A Volcano plot (Fig. 4b) showed the differential expression of DEGs. Furthermore, deregulated genes are shown in Supplementary Fig. 1. Due to the importance of the central genes, the MCOOD method was implemented to identify the hub genes from the network. With attention to the K score = 2, one subnetwork with a 33 node and 455 was recognized, which showed the critical role of 33 genes in CRC (Supplementary Fig. 2).

### Proliferation role of miRNAs in CRC

**Figure 5:**
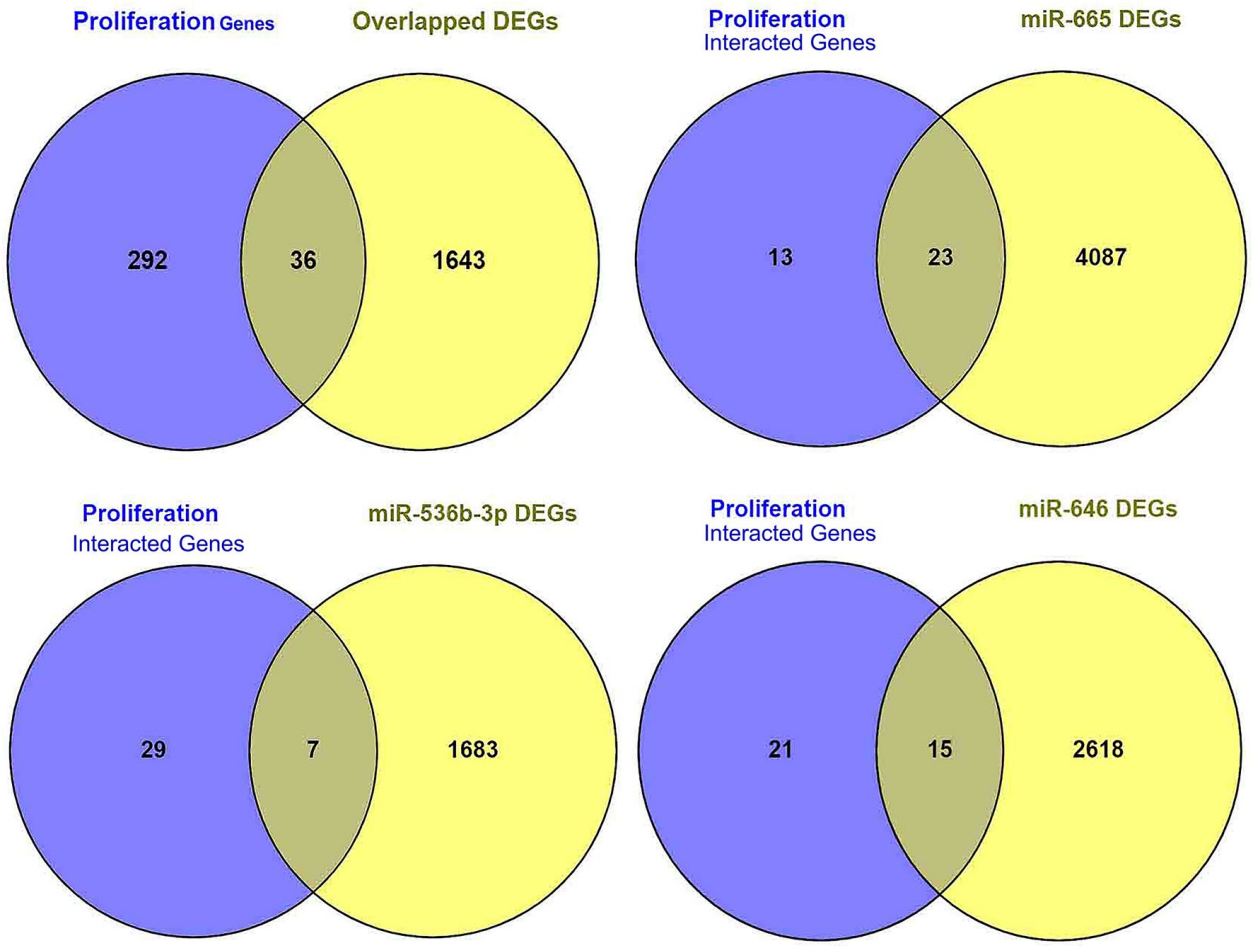
Venn diagram plot of OS genes that are involved in colorectal cancer cell proliferation.

After evaluating the interaction using Venny, we identified 36 genes that contribute to proliferation in CRC. Additionally, we observed that 23 proliferation genes such as ADAMTS8, CADM1, CCL14, CD164, CD24, CD276, CHEK1, CSF1, CTF1, DTYMK, EIF5A2, FGF7, FLT4, FRK, GLP2R, HDGF, IGF1, IL6R, LDOC1, LIF, MCTS1, MDM4, and MKI67 are associated with miR-665, seven proliferation genes such as CSF1, ERBB2, GAB1, GLI2, CXCL8, LIF, and MDM4 are linked to miR-536b-3p, and 15 genes such as BRCA1, CADM1,CDC6, CSF1, CXCL5, FGFBP1, FLT4, FOSL1, GTPBP4, ICOSLG, IL1B, IL6R, KIF2C, LDOC1, and MDM4 contribute to miR-646 Fig. 5.

### KEGG pathway analysis and gene ontology

**Figure 6:**
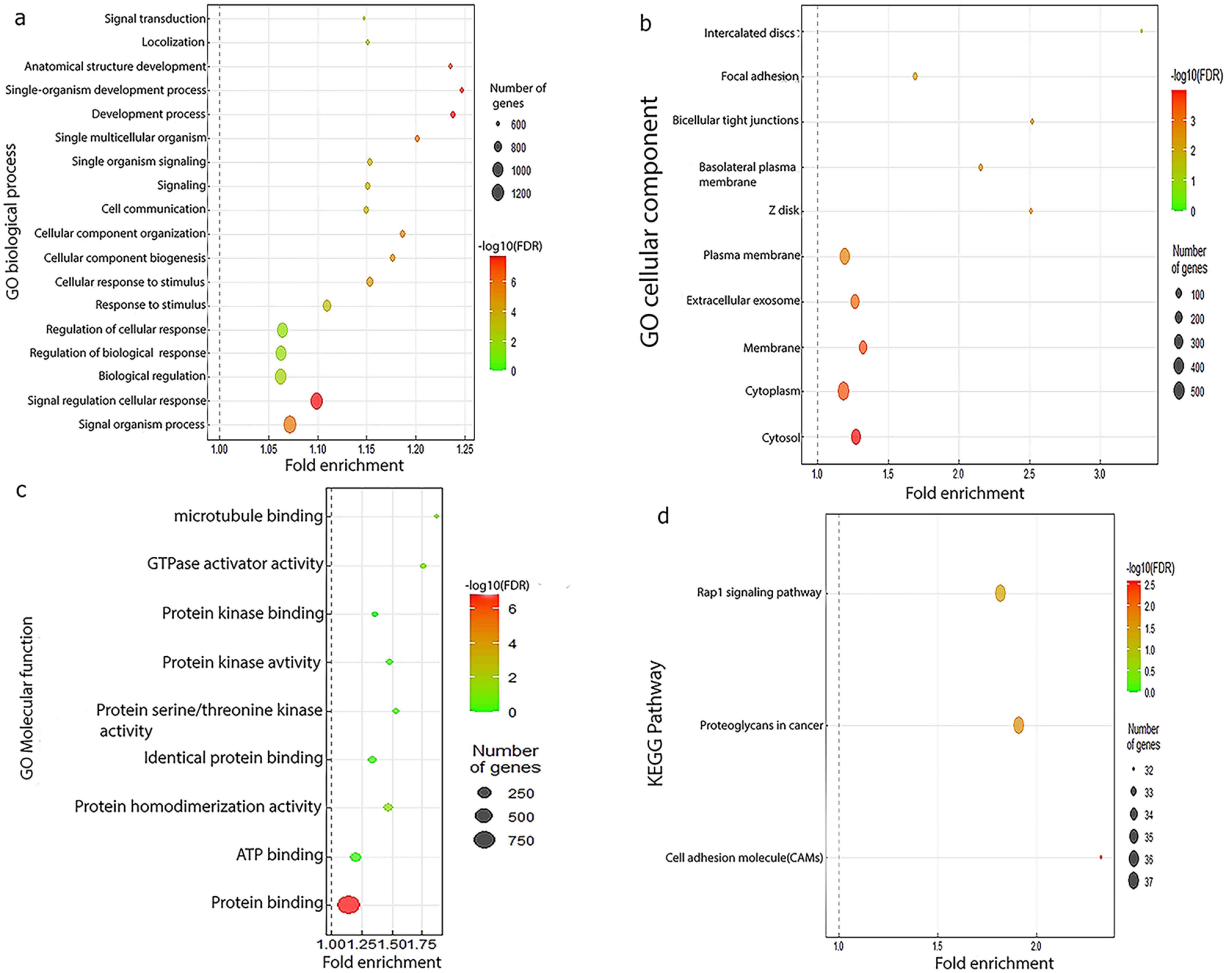
A dot-plot graph of the gene enrichment analysis: **a** analysis of biological processes. **b** analysis of cellular components. **c** analysis of molecular functions. **d** KEGG pathway analysis. The R package 'clusterProfiler' was utilized to conduct GO analysis, and the outcomes were graphically represented using the R package 'ggplot2.'

A KEGG pathway analysis was developed to identify the biological and functional roles of the 1680 identified RNAs and the gene ontology, which is composed of biological processes (BP), molecular functions 16, and cellular components 12. In the case of the Biological process genes, the overlapped genes were associated with cell division, chromatin condensation, nuclear division of mitotic cells, replication of DNA, proliferation of cells, chromatin separation of mitotic cells, the G1/S cell cycle transition, and cellular repair (Fig. 6a). In the case of cellular component^12^, the overlapped genes were related to the cytosol, cytoplasm, plasma membrane, extracellular exosomes, Z disc, basolateral plasma membrane, intracellular tight junctions, and focal adhesion intercalated discs (Fig. 6b). In the case of Molecular Function, the overlapped genes were associated with microtubule binding, GTPase activation, protein kinase activity, and protein binding (Fig. 6c). Based on the analysis of the KEGG, the crucial DEGs were associated with cancer, Rap1 signaling pathways, and cell adhesion molecules (CAMs) (Fig. 6d). Furthermore, we conducted a thorough examination of the functions of the 33 hub genes using gene ontology and KEGG pathway analyses. In terms of biological processes, these central genes were found to be significantly involved in the cell cycle. Regarding cellular components, they exhibited contributions to condensed chromosomes. Molecular functions analysis indicated a strong association with ATP binding. Lastly, the KEGG analysis revealed their involvement in the cell cycle pathway. Furthermore, the gene ontology and KEGG of 33 hub-gene have been evaluated in Supplementary Fig. 3.

### Expression and overall survival validation of hub-genes

**Figure 7:**
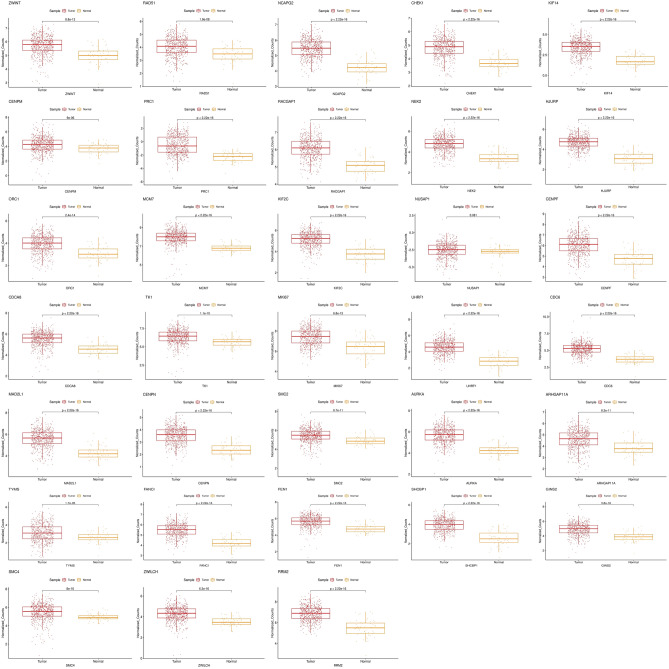
Expression validation of 33 hub-genes using bulk RNA sequencing data. The analysis was performed on samples from a cohort of colorectal cancer (CRC) patients obtained from TCGA.

**Figure 8:**
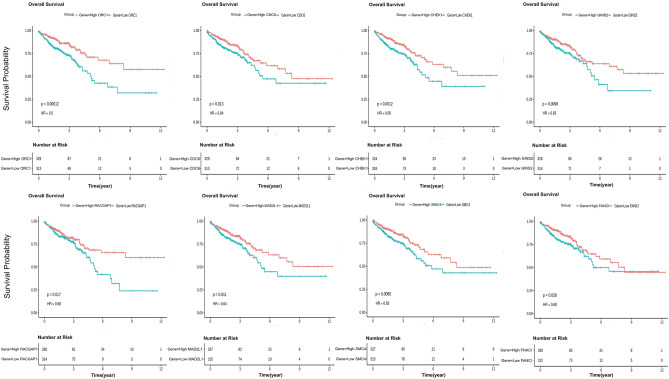
The validation of overall survival genes was conducted using data from TCGA, where 647 samples were categorized into two groups accordance to their expression levels: low and high. Kaplan–Meier curves were generated to illustrate the relationship between the expression of hub genes and the OS of CRC patients. The graph presents the Kaplan–Meier survival curve for the overall survival gene identified in the study.

To comprehensively investigate the roles of the 33 hub genes in CRC, we conducted expression validation using TCGA bulk RNA seq colorectal cancer patient data. Our findings revealed that 32 of these genes exhibit significant overexpression in CRC, as illustrated in Fig. 7. In this study, we performed the overall survival rate on 33 hub genes through TCGA analysis. Only eight genes were identified as having a remarkable impact on the survival outcome of CRC patients. The upregulation of these genes (CHEK1, CDC6, FANCI, GINS2, MAD2L1, ORC1, RACGAP1, and SMC4) can lengthen the Overall survival and can act as a protective factor in CRC patients (Fig. 8).

### Diagnostic and prognostic value evaluation

**Figure 9:**
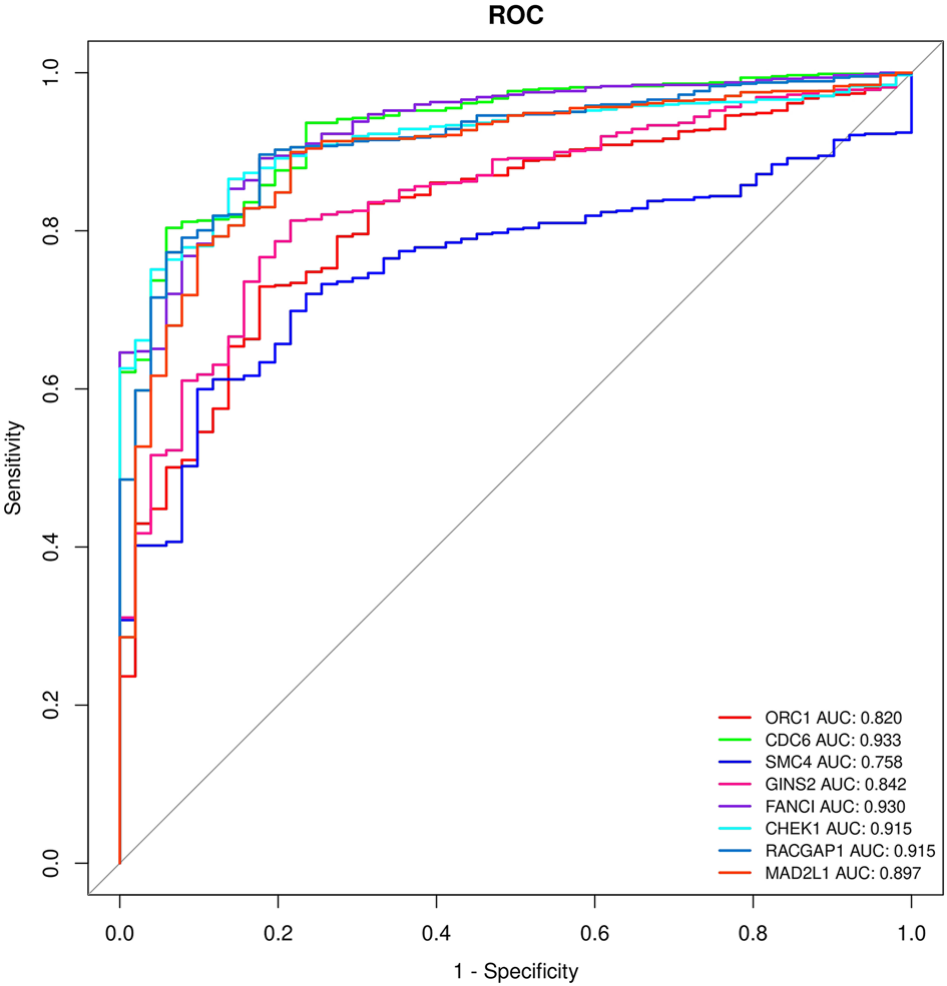
ROC curve analysis of significant OS genes in CRC, presenting individual ROC curves for hub genes based on TCGA data.

In the next step, to investigate the diagnostic value of hub genes, which were significant in overall survival analysis, we applied ROC analysis on TCGA data of colon and rectal cancer—obtained AUC values of hub-genes (ORC1 AUC: 0.820, CDC6 AUC: 0.933, SMC4 AUC: 0.758, GINS2 AUC: 0.842, FANCI AUC: 0.930, CHEK1 AUC: 0.915, RACGAP1 AUC: 0.915, and MAD2L1 AUC: 0.897) proper diagnostic performance of hub genes in colorectal cancer (Fig. 9).

### Gene set enrichment analysis

**Figure 10:**
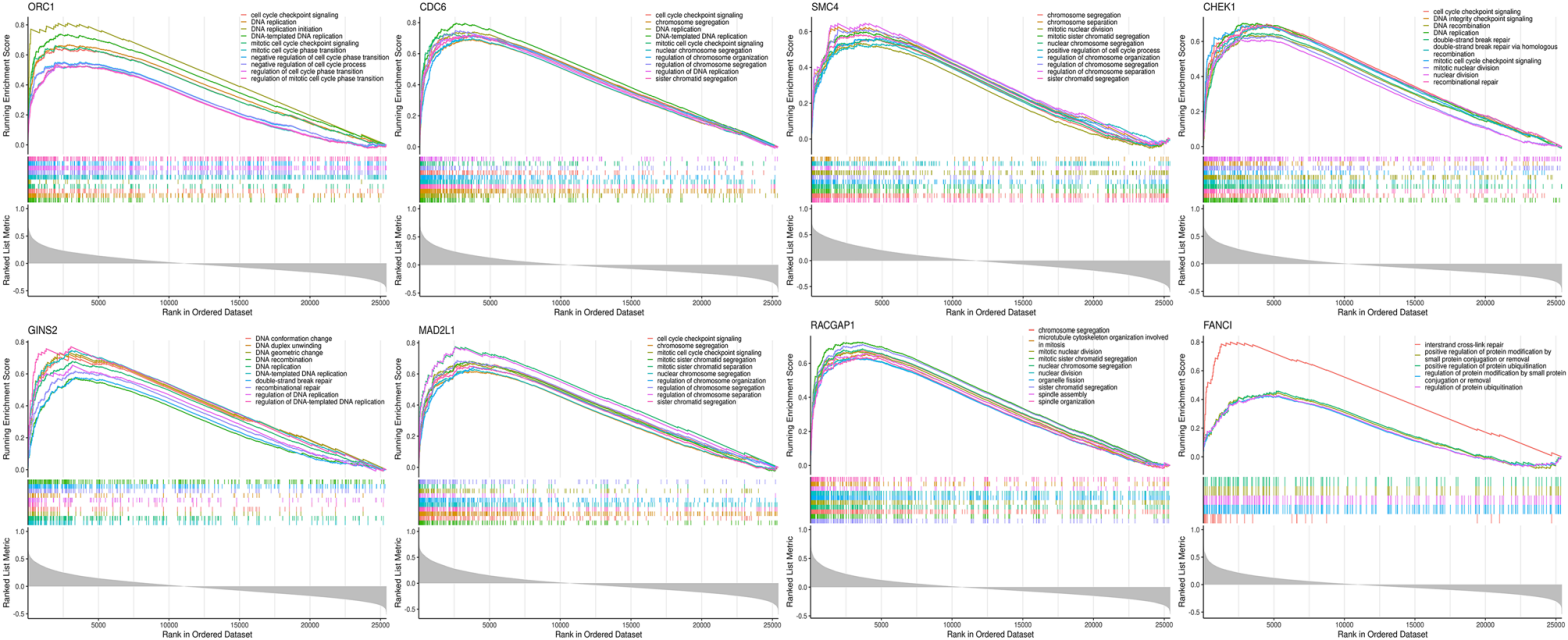
The figure displays the selected results of the GSEA analysis of mRNA data. The panel showcases the pathways that are enriched in the eight-gene group. Gene sets with Normalized Enrichment Score (NES) above 1.0, *p*-value below 0.05, and False Discovery Rate (FDR) q-value under 0.25 were deemed statistically significant in this analysis.

After thoroughly exploring the GSEA of hub-genes, we found that CHEK1, CDC6, FANCI, GINS2, MAD2L1, ORC1, RACGAP1, and SMC4, previously identified as significant central mRNAs, play important roles. The findings demonstrated that the hub genes ORC1, CDC6, CHEK1, and MAD2L1 promoted the cell cycle checkpoint signaling, which is essential in cancer progression. In addition to the mentioned four genes, RACGAP1 and MAD2L1 have critical roles in the regulation of different phases of mitotic division; GSEA analysis also suggested that SMC4 played a role in the separation, segregation, and organization of chromosomes, and RACGAP1 is necessary for chromosome segregation and spindle formation in the cell. Therefore, enriching the hub genes in these pathways suggests that CRC progression will be affected. DNA conformation and geometric changes are controlled under the expression of GINS2, and protein modification and ubiquitination are other biological processes that could be affected by FANCI (Fig. 10).

### Verification of hub genes at the cellular level

**Figure 11:**
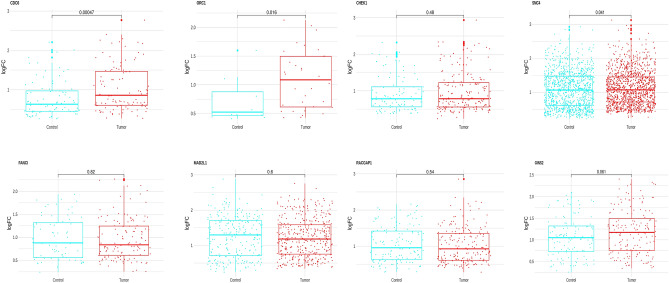
Confirmation of the expression patterns of the eight central genes using scRNA-seq data. Extracted from the GEO database, the scRNA-seq data was analyzed to validate the results in single cells of colorectal cancer (CRC).

scRNA-seq data were used to confirm the expression of the hub genes ORC1, CDC6, MAD2L1, GINS2, FANCI, CHEK1, RACGAP1, and SMC4, in CRC and normal tissue using a single-cell gene sequencing approach. Results significantly confirmed higher gene expression of ORC1 and CDC6 in tumor cells compared to normal cells. In total, 193 cells expressed CDC6, of which 107 were tumor cells and 86 were normal cells. There were 44 ORC1-expressing cells, of which 32 were tumor cells and 12 were normal. Although the number of tumor cells that expressed GINS2 was higher than normal cells, the results were not statistically significant, because of the lower differences in the mean expression of the hub gene between the two cell populations (Fig. 11).

### Contribution hub-genes in known oncogenes and tumor suppressor pathways

**Figure 12:**
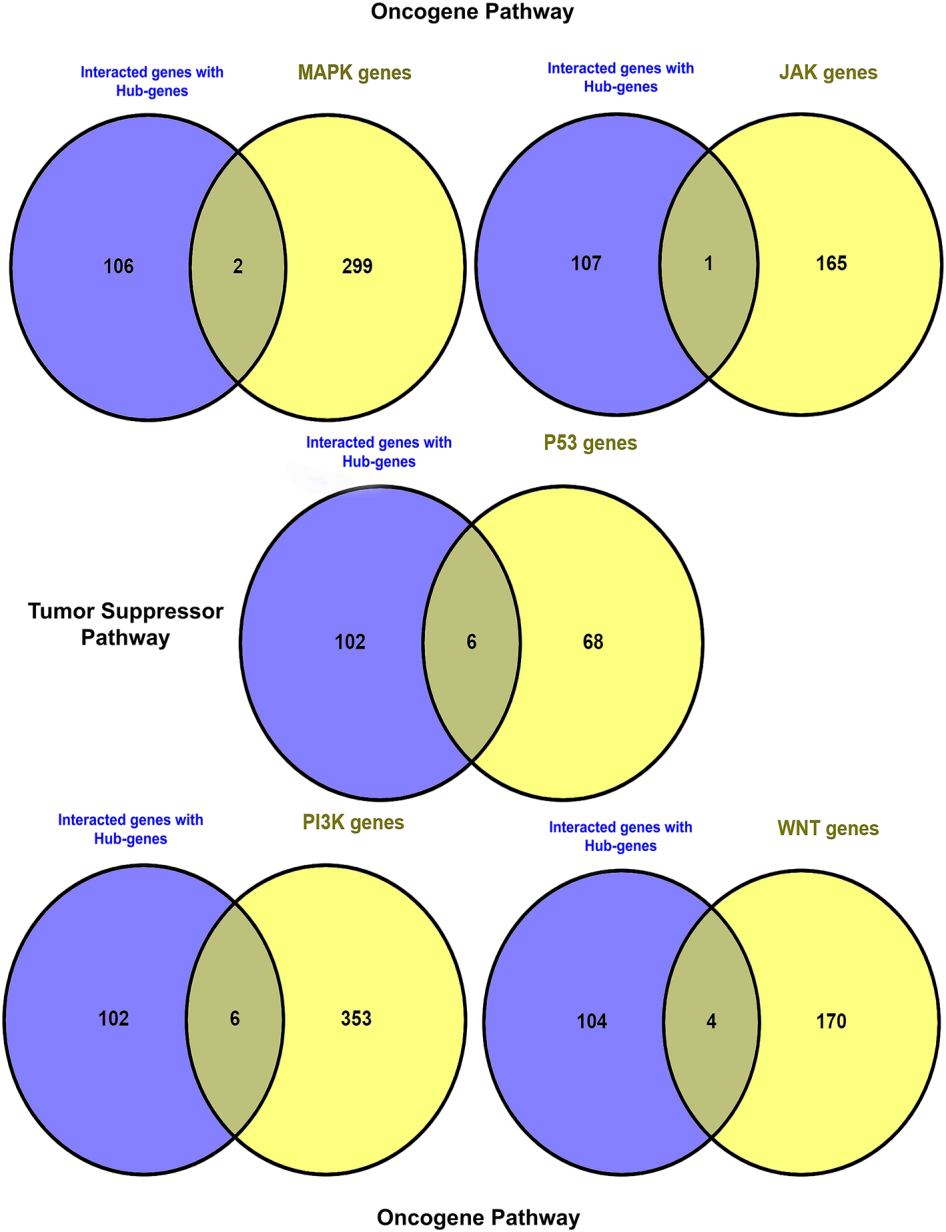
The interaction of eight hub genes with key signaling pathways, including MAPK, JAK-STAT, PI3K-AKT, and P53, which are associated with oncogenic and tumor suppressor functions, respectively.

The eight hub genes interact with various oncogene and tumor suppressor pathways in CRC. A network featuring hub genes and their interacting counterparts is depicted in Supplementary Fig. 4. Following that, we utilized Venny software to analyze the genes that interacted with well-known oncogenes and tumor suppressor genes. Regarding oncogenes, these hub genes have interactions with two genes: TP53 and CDC42, which are part of the MAPK signaling pathway; one gene, CDKN1A, associated with the JAK-STAT pathway; and six genes—PPP2CB, CDK2, TP53, PPP2R2A, PPP2R2B, and CDKN1A—related to the PI3K-AKT oncogenic pathway. Concerning the tumor suppressor pathway, the identified hub genes interact with five genes—CDK2, TP53, CHEK2, CDKN1A, and ATR—involved in the P53 pathway Fig. 12.

### Identification of open chromatin region for CDC6 and ORC1

The CRC samples were categorized into highly expressed and lowly-expressed group sets using the median of CDC6 and ORC1 expression, and the chromatin accessibility landscape was investigated for 23 chromosomes. Differential analysis identified 597 and 351 significant chromatin regions for highly-expressed vs. lowly-expressed CDC6 and ORC1 genes, respectively. The most significant accessible chromatin regions identified during differential accessibility analysis in high levels of CDC6 and ORC1 in comparison to low levels of CDC6 and ORC1 were related to chromosomes 2, 4, and 12. Annotation for these chromatin regions revealed that most of them were distal regions. To visualize the correlation of chromatin regions with samples, a heatmap was generated, and CRC samples were clustered based on the calculated correlation obtained from the count matrix of ATAC-seq. The heatmap also presented the regions with the most variability in accessibility. Peaks were mapped to the human genome for most variable chromatin regions through the coverage plot.

**Figure 13:**
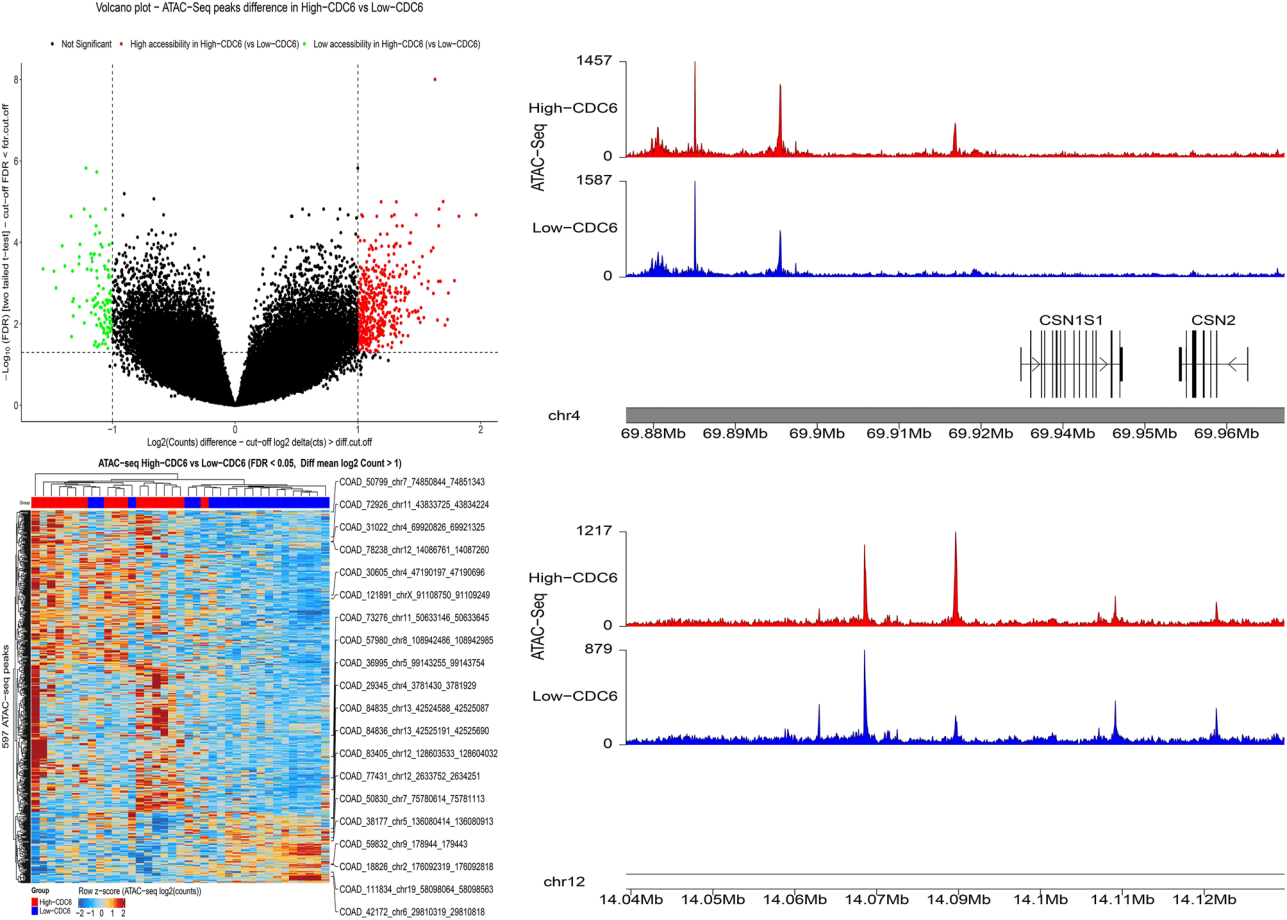
Identification of chromatin accessible regions using ATAC-seq data from TCGA database based on CDC6 expression. Samples were categorized into highly-expressed and lowly-expressed CDC6 samples determined by the central tendency of expression in the target hub gene and the most variable accessible regions were determined with cut-off FDR < 0.05 and |log2 count difference|> 1 as statistical significance level.

**Figure 14:**
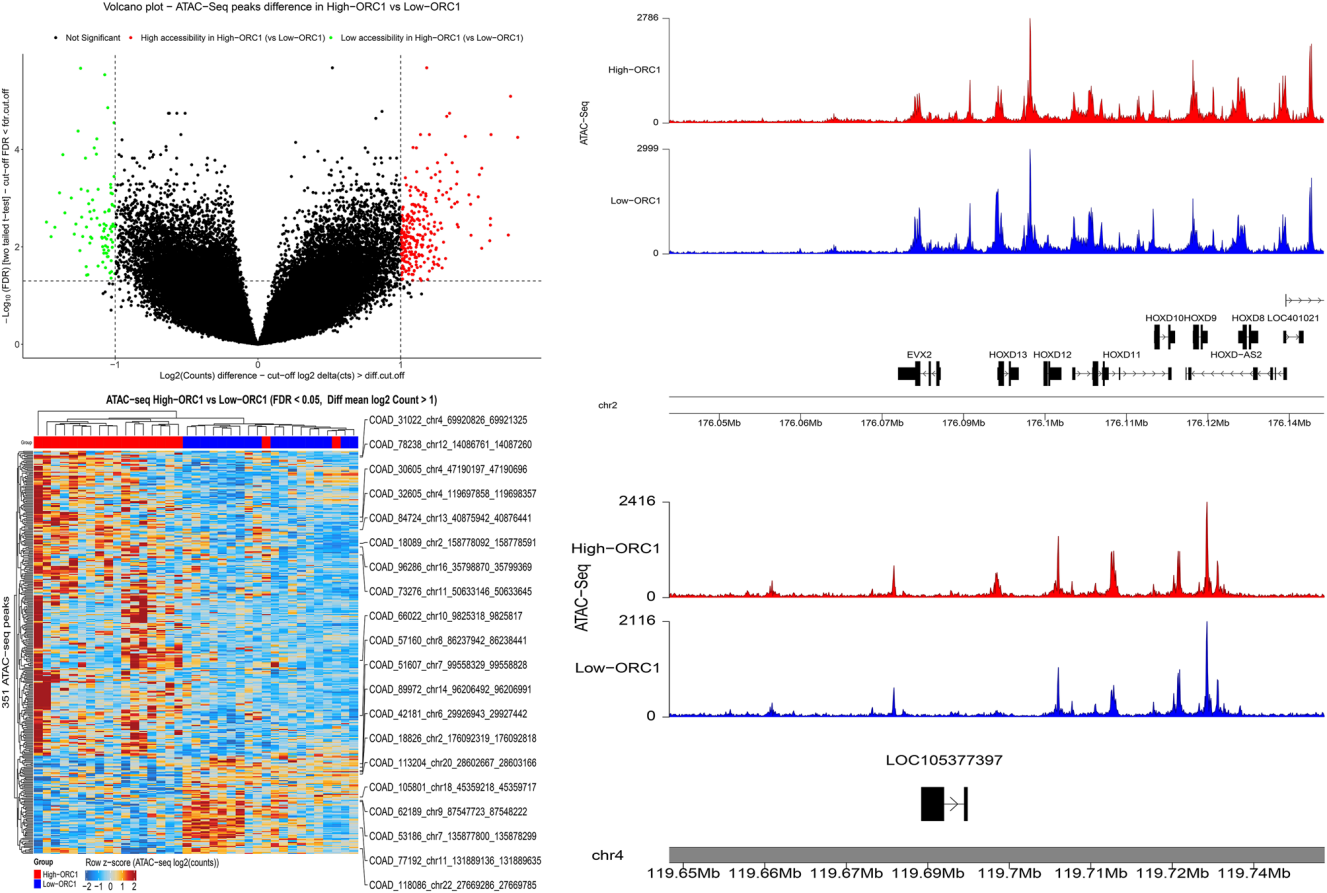
Identification of chromatin accessible regions using ATAC-seq data from TCGA database based on ORC1 expression. Samples were categorized into highly-expressed and lowly-expressed ORC1 samples based on the median of expression of the target hub gene and the most variable accessible regions were determined with cut-off FDR < 0.05 and |log2 count difference|> 1 as statistical significance level.

Chromatin regions that were strongly accessible about the expression of CDC6 and ORC1 were identified for chromosomes 12, 4, and 2. Several stronger significant peaks were observed in the high level of CDC6 than the peaks in the low level of CDC6 in chromosome 12 and chromosome 4. Overall, these results proposed higher accessibility in high levels of the CDC6 gene. Similarly, the sharper peaks in the up-regulation of ORC1 demonstrated a strong correlation with accessibility in Chr2 and Chr4 (Figs. 13 and 14).

### CDC6 and ORC1-induced epigenetic modifications

**Figure 15:**
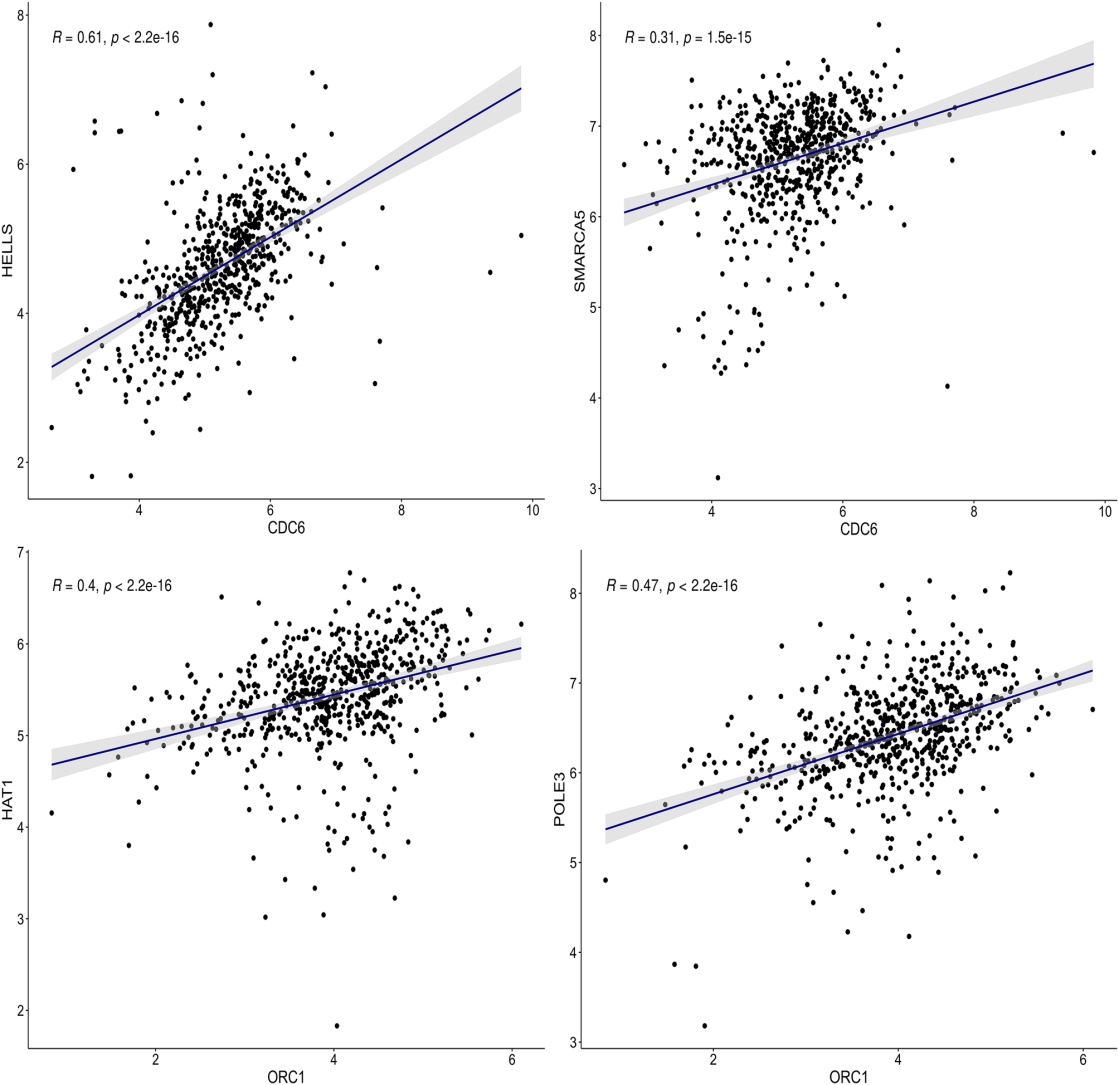
Correlation of CDC6 and ORC1 with other epigenetic elements that regulate chromatin remodeling and histone acetylation.

According to EpiFactor, the CDC6 and ORC1 genes significantly induce epigenetic modifications affecting gene expression in colorectal cancer. Specifically, CDC6 is associated with chromatin remodeling, while histone acetylation is the principal epigenetic modifier driven by the ORC1 gene. To explore the role of CDC6 and ORC1 in epigenetic alterations more precisely, we investigated the connection of CDC6 with chromatin remodeling elements and ORC1 with histone acetylation modifiers by constructing protein–protein interaction networks then 24 first-order neighbor genes for CDC6 and five first-order neighbor genes for ORC1 were extracted to perform correlation analysis. Network edge and Correlation analysis suggested a robust significant association of CDC6 with HELLS and SMARCA5 genes, chromatin remodeling elements. ORC1 was significantly correlated with Histone modifiers HAT1 and POLE3 (Fig. 15).

### Methylation

**Figure 16:**
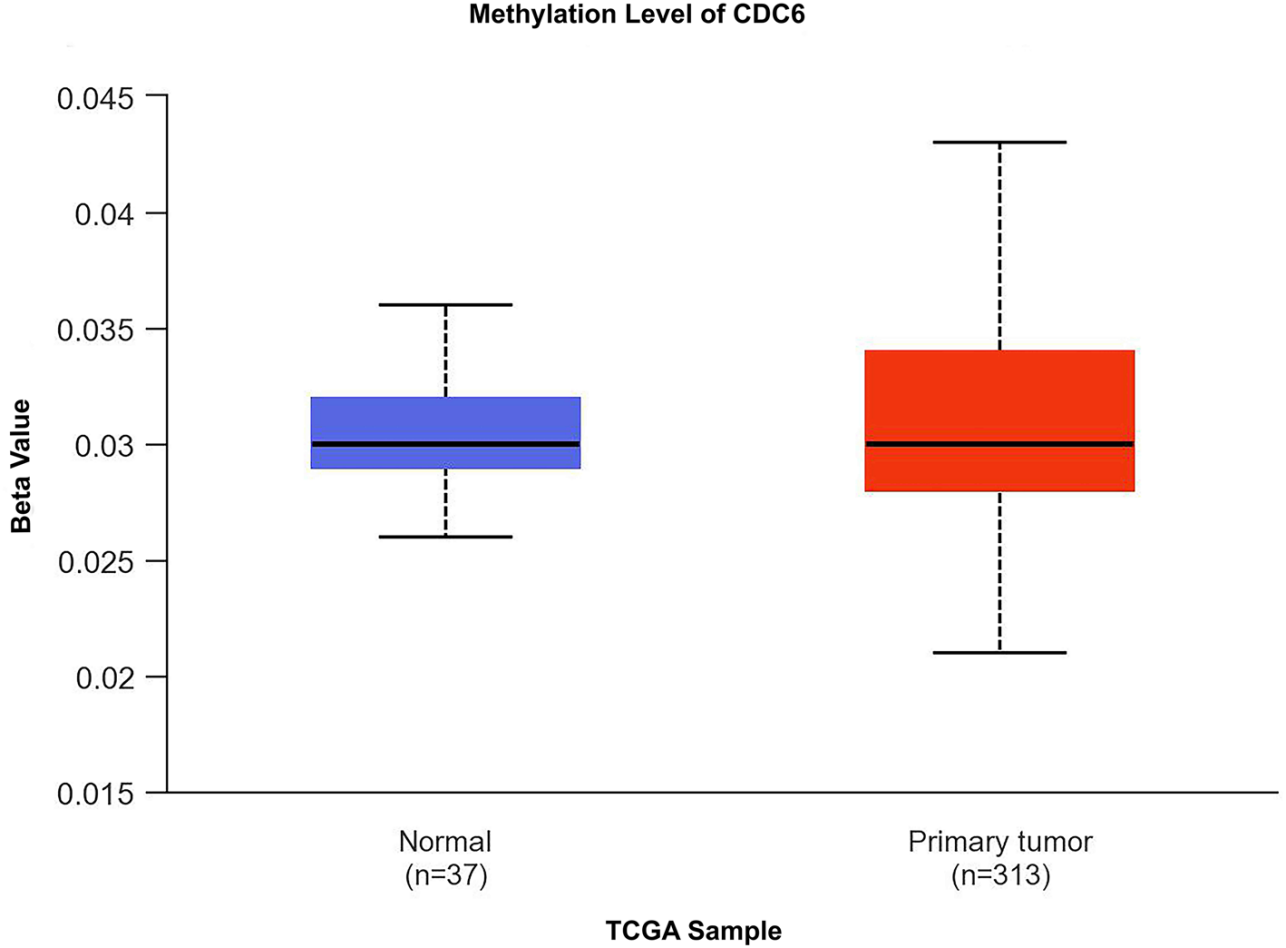
The assessment of CDC6 and its methylation status is depicted in Fig. 13A It displays the promoter methylation level of CDC6 in CRC.

To gain further insights into the methylation behavior of CDC6/ORC1, the present study investigated their methylation status. Interestingly, we observed no significant variation in the promoter methylation level of CDC6 between normal tissues and primary tumor tissues (Fig. 16).

### Characterization of immune features

#### Immune cell infiltration

**Figure 17:**
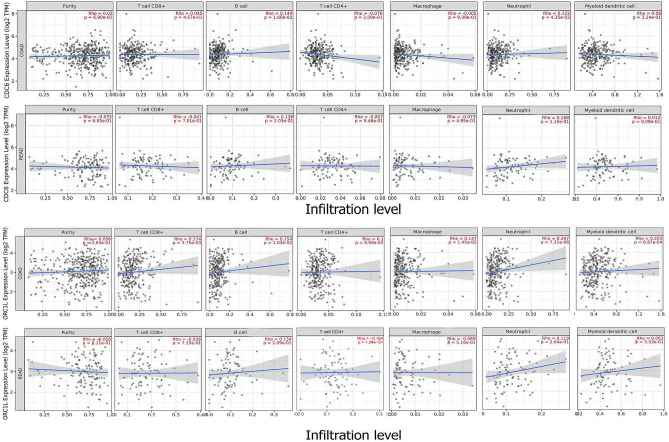
Correlation between CDC6 and ORC1 expression and tumor immune infiltrating cells in colorectal cancer. Panel illustrates the association between the expression of CDC6 and ORC1 and six types of immune cells in colorectal cancer samples.

Utilizing the TIMER database, we explored the expression levels of CDC6 and ORC1 in CRC along with six distinct types of infiltrating immune cells. The results indicated a positive association between CDC6 expression and the presence of CD8^+^ T cells, B cells, neutrophils, and dendritic cells (DCs) in colon adenocarcinoma (COAD). The expression level of ORC1 was a direct correlation with a list of immune cells such as CD8 + T cells, CD4 + T cells, macrophages, B cells, neutrophils, and DCs in COAD. ORC 1 had a direct correlation with B cells, CD4 T cells, neutrophils, and DCs in READ (Fig. 17).

#### Correlation between validated hub genes (CDC6 and ORC1) and immune inhibitors

**Figure 18:**
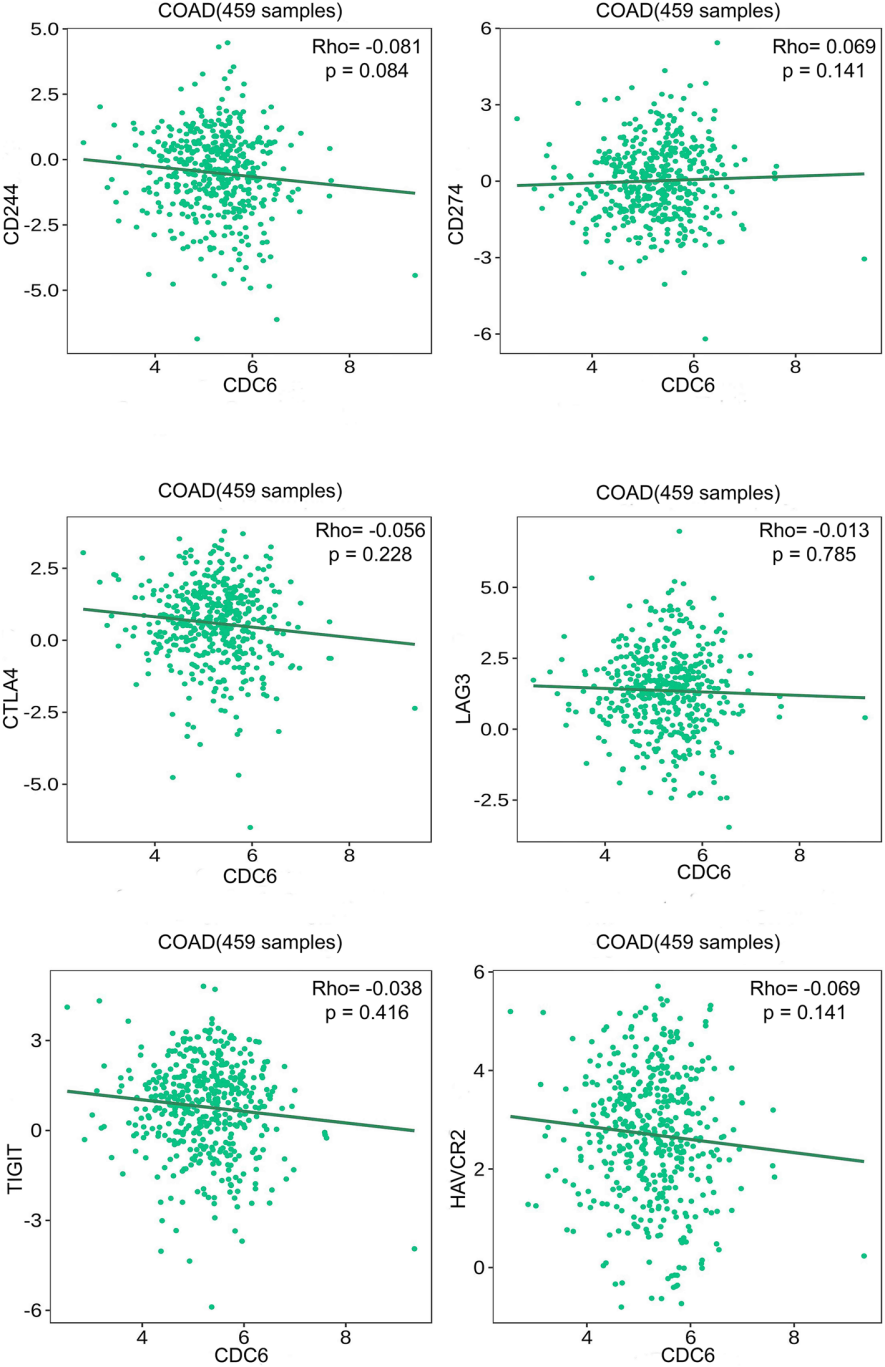
Thorough investigation of CDC6 and diverse immune checkpoint genes using the TISIDB database. The associations of CDC6 with immune checkpoint genes.

**Figure 19:**
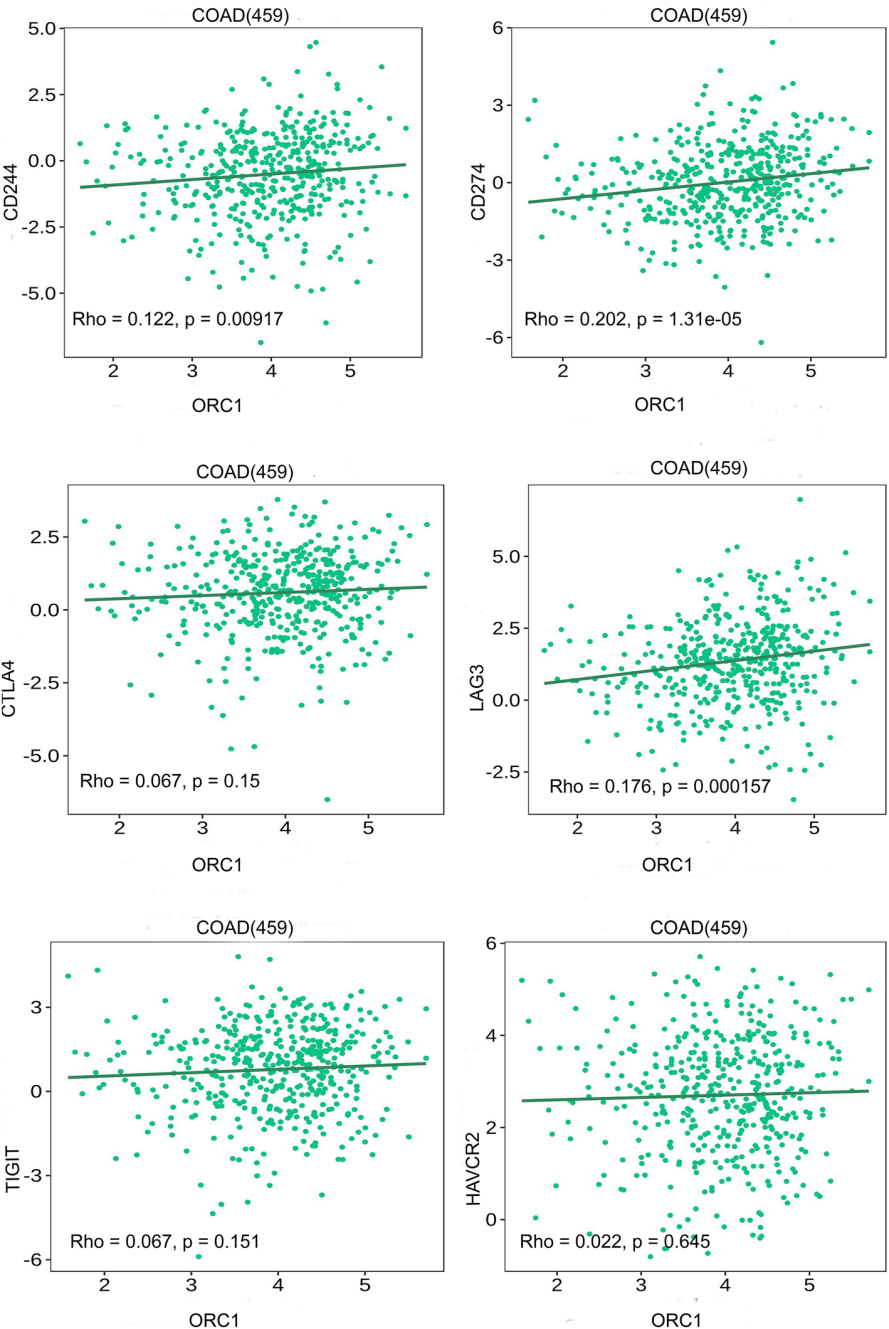
Thorough investigation of ORC1 and diverse immune checkpoint genes using the TISIDB database. The associations of ORC1 with immune checkpoint genes.

A growing body of evidence has substantiated the significance of ICIs as a promising immunotherapy approach with tremendous potential. By utilizing the TISIDB database, we established the relationship between CDC6/ORC1 and more than six crucial ICIs in CRC. We investigated that CDC6 had a significant negative correlation with CD244, CTLA4, LAG3, TIGIT, and HAVCR2, whereas showed a positive correlation with CD274 but was not significant. ORC1 exhibited a significantly positive correlation with CD244, CD274, and LAG3 (Figs. 18 and 19).

#### Prediction of response to ICI therapy in CRC

**Figure 20:**
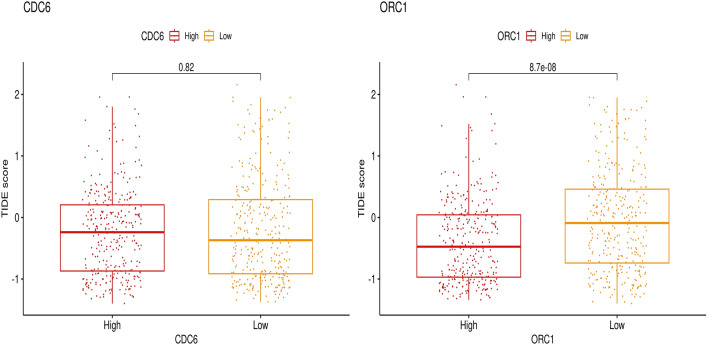
The TIDE score comparison for assessing immunotherapy response in CRC in relation to the expression levels of CDC6 and ORC1.

Colorectal samples in the TCGA cohort were grouped into high-expressed and low-expressed samples based on the median of CDC6 and ORC1 expression, and the Tide algorithm was conducted to investigate immunotherapy response in colorectal cancer samples. Higher expression of CDC6 was associated with higher Tide scores in CRC samples; however the difference was not statistically significant. Tide score was significantly lower in high-expressed ORC1 CRC samples than in low-expressed ones, which proposed a potentially greater response to immunotherapy in CRC samples with higher expression of ORC1 Fig. 20.

## Discussion

A growing body of literature has shown that non-coding RNAs such as circRNAs and miRNAs can act significant functions in several biological processes, especially cancer development and progression. Studies have confirmed that some miRNAs are closely involved in cancer cell migration, proliferation, and invasion. circRNAs may be able to affect these processes by modulating miRNA expression levels in CRC^17–19^. In light of the current research, circRNAs possess the potential to target specific miRNAs, typically referred to as acting as a miRNA sponge or MRE, meanwhile abrogating the miRNA inhibition of their target mRNAs^20,21^. According to these studies, ceRNA can play a pivotal role in the regulation of a wide variety of genes, while some circRNAs could be used as biomarkers for cancer screening and diagnosis. In CRC, some circRNAs, such as circPPP1R12A-73aa and circ100146, are involved in CRC cell proliferation, migration, and invasion, and hence can either promote cancer progression or inhibit tumor suppression effects. Importantly, there are only a few circRNAs so far known to be involved in human cancer, while the majority of circRNAs have so far remained undefined. In the current study, two well-known databases (circ2Trait and circBase) were utilized to evaluate the deregulation of hsa_circ_000240 in CRC. To the best of our knowledge, this is the first time hsa_circ_000240 has been reported to be overexpressed in CRC tumor tissue. In this study, the overexpressed levels of hsa_circ_00240 have been confirmed by qRT-PCR in 31 CRC tissue and corresponding normal tissue pairs.

Galectin 3-binding protein (LGALS3BP), alternatively known as 90K, is a versatile glycoprotein that plays multifaceted roles in both immune responses and cancer-related processes. Given that LGALS3BP serves as the source gene for hsa_circ_000240, it establishes interactions with a diverse range of biological and cellular functions. Notably, these interactions are particularly pronounced in the context of extracellular matrix proteins, ultimately contributing to the advancement of CRC development. Studies on molecular pathways and genetic alterations in colorectal cancer have identified two mutations with COSMIC IDs COSM6700676 and COSM2746802 in gene LGALS3BP which occurred in the genomic region related to hsa_circ_000240 and suggested the potential role of this circular RNA in colorectal cancer. Moreover, Colonic bacteria abundantly release LPS, which interacts with TLR4, initiating the TAK1-NF-κB-cytokine axis and promoting inflammation and colon tumorigenesis. Lgals3bp exerts a negative regulatory role by suppressing TAK1, as evidenced by its upregulation in the inflamed colon with NF-κB activation. The study by Sang-Hee Cho et al. advances our comprehension of colon inflammation regulation, highlighting Lgals3bp's capacity to inhibit TAK1-NF-κB-cytokine signaling, thus preventing excessive inflammation and tumorigenesis. Lgals3bp directly engages with TAK1, reducing protein stability and the affinity between TAK1 and its adaptor proteins^22,23^. This finding establishes Lgals3bp as an endogenous TAK1 negative regulator in the colon, and based on the reference gene hypothesis, suggests that over-expression hsa_circ_000240 might serve as a potential therapeutic or critical role in colorectal cancer development by influencing TAK1-NF-κB signaling pathways.

The ceRNA hypothesis is a theory that non-coding RNAs (such as circRNAs, lnc-RNAs, miRNAs, and some pseudogenes) can control each other’s expression by competing for the binding to certain shared MREs^24^. In the present study, circ000240 was found to be a ceRNA to make bonds of three different miRNAs (miR-646, miR-665, and miR-526b-3p) based on the *CircInteractome* (Table 1)*.* Studies have shown that miRNAs can interact with the 3′-UTR or 5′-UTR of the interest RNAs by complementary base-pairing and can then decrease or overexpress the expression of target genes at the post-translational level. However, no study to date has found any relationship between circ000240 and the three detected miRNAs, hsa-mir-646, hsa-mir-665, and hsa-mir-526b-3p. In other studies, a correlation between the three above-mentioned miRNAs and other different circRNAs has been reported. For example, hsa_circ_0074241 could bind competitively to miR-646, and then act as an RNA sponge for the regulation of the expression of EGFR (epidermal growth factor receptor). Indeed, they suggested that the hsa_circ_0074241 interaction with EGF signaling could affect the production of ECM (extracellular matrix) by directly regulating miRNA-646^25^. circ_0044556 acted as an oncogene in CRC by affecting the miR-665/DIAPH1 (diaphanous homolog 1) pathway^26^. Circ_0101802 participated in CRC progression by modulating the miR-665/DVL3 (disheveled 3) signaling pathway^27^. miR-646 reduced the expression of Nin one binding protein (NOB1) to suppress the growth of CRC cell lines^28^. Through a comprehensive analysis of GO proliferation genes and their shared miRNA targets within the pool of DEGs in CRC, we have unveiled connections between hsa-mir-646, hsa-mir-665, and hsa-mir-526b-3p and the regulation of cell proliferation in CRC. Notably, the elevated expression of ICOSLG has emerged as a significant driver of proliferation in CRC^29^, which is influenced by the action of miR-646. This underscores the pivotal role of miR-646 in CRC development, particularly in promoting cell proliferation. To the best of our knowledge, no reports have shown that hsa_circ_000240 can act as a sponge for miR-665. These reports suggest that the detected miRNAs may play a crucial role in cancer therapy. According to the latest literature, no studies have reported that hsa_circ_000240 can act as a sponge for miR-526-3p in CRC or other cancers.

By examining the downstream targets of miRNAs, our KEGG pathway analysis revealed that miR-665 significantly affects the CAMs pathway, miR-526b-3p significantly influences the Calcium Signaling pathway, and cGMP-PKG signaling pathway, and finally, miR-646 significantly contribute to MAPK signaling pathway.

To investigate the relationship of circRNA 000,240 on the expression of mRNAs, 1680 overlapping genes (obtained from miRwalk and TCGA database) were used to perform a gene-enrichment analysis. These shared genes exhibited significant enrichment in critical biological processes, including cellular proliferation and transcriptional regulation. KEGG pathways indicated that these genes demonstrated remarkable enrichment in the Rap1 signaling pathway, cancer proteoglycans, and CAMs. Additionally, these pathways are involved in different cancer types. It is proposed that hsa_circ_000240 may affect gene expression via several miRNA/mRNA interactions.

Rap1 is a small GTPase that regulates various processes such as cell junctions and adhesion, and signal transduction. Rap1 is activated by tyrosine kinases (such as PKA and PKC) as well as upstream signaling mediators (such as cAMP and calmodulin). Some downstream interacting molecules, such as the proto-oncogene B-Raf can regulate the expression of genes involved in cell adhesion, proliferation, etc^30,31^. Tsygankova and her team demonstrated that in CRC, Rap1GAP restrains the spreading and migration of cells on collagen IV. In an independent investigation, Kim and his research group found that diminishing Rap1GAP levels in tumor cells amplifies their aggressiveness within the context of CRC^32^. These findings collectively emphasize the significance of the RAP1 signaling pathway in influencing the development and pathogenesis of CRC.

CAMs play a pivotal role in the metastatic potential of CRC. The downregulation of cadherins and catenins facilitates the detachment of tumor cells from the primary site. Concurrently, the selective expression of cell adhesion molecules on different organs and endothelia, in conjunction with the presence of dissimilar adhesion ligands on various colorectal cancer cell lines^16^, suggests that based on the source gene (LGALS3BP) our interested circRNA may also mediate the selection of the host organ for the development of distant colorectal metastases, further underscoring their significance in CRC pathogenesis.

In the case of cancer proteoglycans, they can stimulate chemokines, growth factors, and affect co-operative signaling pathways. The effect of proteoglycans on the tumor microenvironment has been reported both in solid tumors and blood malignancies. Importantly, some cancer immunology and immunotherapy approaches have used proteoglycans as targets for treatment^33^. The intersecting genes, whose modulation or regulation is influenced by circRNAs, hold a crucial significance in the signaling cascades of CRC.

In this research, a list of 33 central genes was found PPI network (Fig. 6). Furthermore, we evaluated the expression levels of hub genes and we demonstrated 32 hub genes have significant expression in CRC and investigated the relationship between hub genes and overall survival in patients using RNA seq data from TCGA on over 600 CRC patient cases. Among the 33 hub genes, eight of them, namely CHEK1, CDC6, FANCI, GINS2, MAD2L1, ORC1, RACGAP1, and SMC4, significantly influence the overall survival of CRC patients as well. Through an analysis of the ROC curve and survival data, we have identified eight genes that exhibit significant associations with overall survival and demonstrate high AUC rates in CRC. Notably, CDC6, FANCI, CHEK1, and RACGAP1 exhibit AUC values of 0.933, 0.930, 0.915, and 0.915, respectively.

CHEK1 is a crucial regulator of cell cycle checkpoints. It is activated in response to DNA damage, replication stress, or incomplete DNA replication. In CRC, CHEK1 is often upregulated, and its overexpression contributes to chemoresistance. When DNA damage occurs, CHEK1 phosphorylates downstream effectors, such as CDC25A, leading to cell cycle arrest to allow for DNA repair. However, in CRC, overexpression of CHEK1 can lead to the evasion of cell cycle checkpoints, allowing damaged cells to continue dividing, which promotes tumorigenesis^34^. Therefore, a potential strategy for managing CRC is the utilization of a combination approach involving a CHK1 inhibitor along with conventional chemotherapy agents, which could prove to be an effective method in overcoming chemoresistance.

RACGAP1, or Rac GTPase-activating protein 1, plays a crucial role in CRC progression. Its heightened expression in CRC tissues is linked to aggressive tumor behavior and poor patient prognosis. RACGAP1 interacts with Rho GTPases like Rac1 and Cdc42, influencing cytoskeletal dynamics and cell motility^35^. This protein promotes cell migration and invasion, critical steps in metastasis, making it a potential biomarker for disease aggressiveness. Targeting RACGAP1 presents a promising approach to hinder CRC metastasis, offering hope for more effective interventions in this challenging disease.

Interventions targeting CHEK1, CDC6, and MAD2L1 pathways offer promising avenues for modulating CRC cell proliferation and survival. CHEK1, as a regulator of the cell cycle and DNA damage response, can be inhibited to disrupt cell cycle progression and sensitize CRC cells to DNA-damaging agents, potentially curtailing proliferation and enhancing cell death. CDC6's essential role in DNA replication initiation can be exploited to hinder CRC cell growth by impairing DNA replication processes. Meanwhile, MAD2L1's involvement in the spindle assembly checkpoint during mitosis makes it a potential target to disrupt proper chromosome segregation and cell division, influencing CRC cell survival and genomic stability. These targeted interventions hold therapeutic potential, warranting further investigation and clinical exploration in the context of CRC treatment.

We conducted an overall survival analysis on TCGA data to estimate the prognosis value of hub genes in colorectal cancer. Cox proportional hazard regression identified the hazard ratio for the hub genes. ORC1, CDC6, SMC4, GINS2, FANCI, CHEK1, RACGAP1, and MAD2L1 were statistically significant in overall survival analysis, and the hazard ratio of less than 1 presented genes as beneficial factors in colorectal cancer prognosis. ROC analysis of these eight hub genes determined AUC and validated the diagnostic value of hub genes. Early detection and precise assessment of prognosis are crucial for enhancing the survival rates of individuals with colorectal cancer. Consequently, these eight central genes possibly act as potential significant diagnostic markers and predictive indicators for prognosis in colorectal cancer.

Our gene ontology enrichment analysis of the 33 hub genes unveiled their significant roles in processes associated with the cell cycle, chromosome function, and ATP binding. Furthermore, KEGG pathway analysis indicated their active participation in the cell cycle pathway. As per the DAVID disease analysis, most of the hub genes—AURKA, FEN1, MKI67, NUSAP1, RRM2, TK1, and TYMS—were identified as contributors to colorectal cancer progression, as detailed in Supplementary Excel 2.

In this section, we provide a concise overview of the involvement of select hub genes in the development of CRC. Recent research has unveiled intriguing insights, particularly concerning AURKA. It has been observed that AURKA exerts its influence by suppressing the expression of several DNA damage repair genes in a TP53-dependent manner, thereby inhibiting the DNA damage response. This phenomenon may, in part, account for the association between AURKA and a favorable outcome in CRC^36^.

Recent data has unveiled a noteworthy disparity in the expression of MAD2L1 between CRC tissues and normal tissues, with CRC tissues exhibiting higher levels. Remarkably, when MAD2L1 was selectively silenced, a significant inhibition of CRC cell growth was observed. This inhibition was attributed to the disruption of cell cycle progression and the induction of apoptosis in these cells^37^. In CRC, increased MAD2L1 expression can lead to mitotic defects, aneuploidy, and genomic instability. This can result in the survival and proliferation of cells with abnormal chromosome numbers, contributing to tumor progression. Furthermore, the expression levels of these eight hub genes were found to be significantly associated with CRC progression, underscoring their importance in the disease.

To comprehend the significance of hub genes in well-known oncogenes and tumor suppressor pathways, we conducted a network analysis. Our findings revealed that the identified hub genes play a crucial role in oncogenic and tumor suppressor pathways like Wnt, MAPK, JAK-STAT, and P53.

The Wnt signaling pathway represents an intricate web of protein interactions that primarily come into play during embryonic development and cancer, yet it also exerts influence over normal physiological processes in adults. Within the domain of Wnt signaling, the pivotal events center around the alteration and breakdown of β-catenin, a functional actor, impacting both the pathway's functionality and the advancement of colon cancer. Consequently, the Wnt signaling pathway emerges as an essential player in the genesis of diseases, with a particular emphasis on its role in the pathogenesis of CRC^38^. In our study, we found that our hub genes interact with four genes, namely TP53, RBX1, CUL1, and RHOA, all of which are components of the Wnt signaling pathway. This outcome underscores the significant role these hub genes play in the progression of CRC through the Wnt pathway.

The MAPK pathways are signaling pathways that respond to various growth-factor receptors, including the one responsible for epidermal growth factor. In CRC, it's often observed that the epidermal growth factor receptor is excessively produced and activated. There is substantial evidence suggesting that the overexpression and activation of ERK MAPK, a key component of these pathways, play a crucial role in driving the progression of CRC. These changes can involve mutations in various genes, such as APC, KRAS, and BRAF, which are known to be part of the MAPK pathway. Consequently, targeting ERK MAPK has emerged as a promising molecular approach for treating this disease. In our present study, these core genes interact with TP53 and CDC42, both of which are components of the MAPK signaling pathway. This evidence highlights the involvement of these hub genes in the CRC pathogenesis.

The JAK/STAT signal transduction pathway serves as a swift channel for conveying extracellular signals directly to the nucleus. In the context of colon cancer, this pathway assumes a crucial role in rapidly activating oncogenes, which are genes that can drive the uncontrolled growth of cancer cells, and simultaneously inactivating tumor suppressor genes, which normally help cancer development. The identified hub genes are connected to CDKN1A, a constituent of the JAK-STAT pathway. In light of the JAK-STAT pathway, these hub genes play crucial roles in the progression of CRC.

The transcription factor p53 serves as a pivotal tumor suppressor by coordinating a wide range of cellular responses. These responses include DNA repair, halting the cell cycle, inducing cellular senescence, triggering cell death, promoting cell differentiation, and regulating cellular metabolism. Mutations in p53 in the context of cancer not only disrupt the mutant p53's capacity to activate typical p53 target genes but can also imbue it with new oncogenic properties that contribute to the process of tumorigenesis. P53 stands out as the most frequently mutated gene in human tumors. Among all cancer types, colorectal cancer takes the lead with the highest incidence of p53 mutations, affecting approximately 43% of CRC cases (according to data from the IARC TP53 database, R20; accessed on 1 April 2021 at https://p53.iarc.fr/TP53SomaticMutations.aspx). The identified hub genes interact with five genes—CDK2, TP53, CHEK2, CDKN1A, and ATR—involved in the P53 pathway^40^. Furthermore, our identified hub gene is associated with P53, a well-known tumor suppressor gene, suggesting that these hub genes may hold clinical promise in the development of novel immunotherapy drugs for CRC. Targeting these hub genes is anticipated to offer innovative treatment approaches for CRC.

Based on GSEA analysis, a majority of the overexpressed genes demonstrated a significant contribution to regulating the cell cycle, DNA replication, and repair, as well as protein ubiquitination. Our results indicate that these upregulated core genes play a pivotal role in cancer drug development and the management of CRC and other malignancies.

With the recent progress in genomics, single-cell level data analysis has provided us with the ability to examine genetic complexity within tumor tissue at a cellular level. As conventional cancer treatments are often accompanied by drug resistance and side effects, this technological advancement aids in the design of more effective drugs. In this study, we extracted independent single-cell data for colorectal cancer from the GEO database to further investigate at the cellular level. Our analysis revealed significant expression of hub genes, particularly ORC1 and CDC6 genes, which could potentially be used in drug development or as a biomarker in clinical settings. In summary, CDC6 (cell division cycle 6) plays a pivotal role in DNA replication and has been implicated in carcinogenesis. There is some evidence that CDC6 can repress the tumor suppressor *DH1* (E-cadherin), which influences the epithelial-to-mesenchymal cell transition (EMT). It is known that gene amplification has a significant role in the development of tumors^41^. Cheng et al. demonstrated that CDC6 was over-expressed in CRC, and a low expression of CDC6 can inhibit cell growth^42^. To the best of our knowledge regarding CDC6, the ceRNA pathway of hsa_circ_000240/miR-646/CDC6 has not been investigated in oncology. ORC 1 enhances the malignant behavior of lung cancer cells by targeting the Wnt signaling pathway^43^. The ceRNA interaction between hsa_circ_000240/miR-646/ORC1 has not been reported yet in published research.

TCGA RNA seq data and independent scRNA-seq data confirmed CDC6 and ORC1 as potential hub genes in CRC patients. In the next step, transcriptomic data was integrated with ATAC-seq data to obtain deeper insight into the role of CDC6 and ORC1 at the epigenomic level. Differential accessible analysis identified regions on chromosomes 12, 4, and 2 as more accessible regions in CRC patients with high levels of CDC6 and ORC1. The chromatin region on chromosome 12 from nucleotide 14,086,761 to nucleotide 14,087,260 was a significant accessible chromatin region in high levels of CDC6 and ORC1 and data extracted from the Xena browser (https://xenabrowser.net/datapages/) suggested linkage of peak related to this distal chromatin region with genes such as GRIN2B, GNAI2P1, RPL30P11, ATF7IP, PLBD1, RP11-502N13.2, RN7SKP134, PLBD1-AS1 and others. Based on recent research, GNAI2P1 mutations impaired its tumor suppressive function and promoted tumor progression. Moreover, in this annotation, some genes have not been reported yet, so it will be a potential candidate for investigation of their role such as RPL30P11, RNU6-491P, AC007535.1, etc.

Chromatin remodeler proteins act like gatekeepers, controlling access to DNA within nucleosomes. They play a crucial role in allowing various biological functions to happen. When these proteins are expressed incorrectly or undergo epigenetic changes, cancer cells gain the ability to alter their genetic code, maintaining traits that promote cancer growth. In essence, cancer cells can choose specific sets of chromatin remodeler proteins to give them an advantage in promoting cancerous behaviors^44^. Based on recent literature on chromatin modeling's role in CRC development and liver metastasis^45^, our findings indicate that the chromatin remodeling function of CDC6 is likely to play a crucial role in epigenetic regulation.

Apart from DNA methylation, miRNA levels, and genomic imprinting, histone modification is increasingly acknowledged as a vital mechanism that underlies the initiation and progression of colorectal cancer. The improper regulation of histone modification, such as alterations in acetylation, methylation, and phosphorylation levels at specific residues, has been implicated in a broad range of cancers, including colorectal cancer. Furthermore, given that this process is reversible and involves a multitude of dysregulated enzymes, targeting the activity of these histone-modifying enzymes and regulating their levels has been considered a potential avenue for cancer therapy. Based on recent findings, our research has demonstrated the significant importance of the histone modification function of ORC1 for epigenetic regulation^46^. Crucially, during the G1 phase, the minichromosome maintenance complex is recruited to ORC-binding sites through the involvement of CDC6 and CDT1, leading to the formation of a pre-replication complex^47^. The N-terminus of CDC6, along with the HP1 chromoshadow domain^48^, forms a complex, and any modifications, such as chromatin remodeling or acetylation in CDC6/ORC1, can result in delayed DNA replication and promote cancer, such as colorectal cancer.

On the other side, CDC6 has positive correlations and interacts with HELLS and SMARCA5, which regulate the transcriptionally accessible state of chromatin. HELLS is a chromatin remodeling ATPase that provides chromatin-accessible regions for methyltransferase enzymes and affects the expression of various genes in cancer cells. Up-regulation of HELLS in CRC promotes the proliferation and migration of cancer cells, and HELLS knockdown induces G2/M arrest in CRC^49^. Investigation of the epigenetic regulation role of ORC1 identified a positive significant correlation with histone modifiers HAT1 and POLE3. Studies have established the role of POLE3 as a histone-fold protein that interacts with CHRAC1, contributes to DNA repair, and prevents DNA damage in CRC^50^. This investigation has revealed the significant impact of epigenetic changes following the expression of CDC6 and ORC1, highlighting their potential as novel therapeutic targets in cancer treatment.

In our investigation, we observed an upregulation in the promoter methylation level of CDC6 in primary tumors. However, this methylation level remained in the hypomethylated range, failing to fully elucidate the alteration of CDC6 expression. Furthermore, the ceRNA regulatory axis related to these significant over-expressed genes at the cellular level such as ORC1 and CDC6 was constructed. This network might help cancer scientists to better understand biological interactions and could allow more research on colorectal cancer. To comprehensively assess the connection between immune cell infiltration and CRC samples, we performed an analysis to investigate the alignment between the expression levels of CDC6 and ORC1 and the various immune cell types. It is possible that these cells contributed to the complex tumor microenvironment, potentially promoting tumor progression in CRC, despite the idea that they were attacking the tumor. Our analysis revealed that we observed a positive association between CDC6 expression levels and the infiltration of B cells, neutrophils, and myeloid dendritic cells in COAD and READ. Similarly, ORC1 expression levels were positively correlated with the infiltration of B cells, CD8 + T cells (COAD), neutrophils, CD4 + T cells (COAD), macrophages (COAD), and myeloid dendritic cells (COAD and READ). These findings suggest that these genes play a crucial role in shaping the CRC tumor microenvironment and could potentially have an impact on the effectiveness of immunotherapy.

Furthermore, in this study, we investigated the relationship between CDC6/ORC1 and immune checkpoints. Our findings revealed that ORC1 exhibited a strong positive correlation with CD244, CD274, and LAG3. Overall, ORC1 is a crucial gene closely linked to CD274 and LAG3 in CRC and holds promise as a potential therapeutic target.

The Tide algorithm showed that patients with higher expression of ORC1 had lower T cell dysfunction and higher T cell infiltration into the tumor environment. This indicates a potentially more effective response to immune checkpoint blockade therapy. ORC1 could be a potential biomarker for ICI immunotherapy or a novel treatment target for CRC.

Regarding CDC6, we observed no significant change in the TIDE score between high and low CDC6 expression levels, suggesting that it may not play a critical role in CRC ICI therapy. However, it's important to note that the literature highlights CDC6 as a pivotal molecule involved in DNA replication and its transitional effects on EMT. Specifically, CDC6 is known to significantly impact cancer invasiveness by repressing the regulation of E-cadherin. Mechanistically, the overexpressed CDC6 binds to an E-box motif within the promoter region, displacing the chromosomal insulator CTCF and consequently silencing E-cadherin. This dual function can activate DNA replication origins while simultaneously leading to transcriptional repression, serving as a "molecular switch." Given these findings, CDC6 holds potential as a therapeutic target, particularly in the context of CRC and cancer therapy.

Finally, the multifaceted pathogenesis of CRC is influenced by a myriad of factors, including miRNAs such as miR-646, miR-665, and miR-526b-3p, which regulate gene expression and form complex ceRNA networks with circRNAs like hsa_circ_000240, impacting the regulation of target genes, and interacting with hub genes like CDC6 and ORC1 vital for DNA replication and epigenetic regulation. In addition to the genetic landscape, chromatin accessibility and epigenetic modifications, mainly conducted by CDC6 and ORC1, play crucial roles in CRC progression, possibly affecting gene regulation and pathway control. Furthermore, the association of genes like ORC1 with immune checkpoint genes (e.g., CD274 and LAG3) integrates CRC biology with the immune microenvironment, suggesting that multi-modal therapeutic approaches targeting genetic, epigenetic, and immune facets may offer a holistic and potentially more effective treatment strategy against CRC.

## Conclusion

Utilizing a diverse array of genomics tools, including qRT-PCR, RNA-seq, scRNA-seq, ATAC-seq, and methylation analysis, we have successfully established an hsa_circ_000240-associated competing endogenous RNA (ceRNA) network, shedding new light on the mechanisms underlying CRC. We recognized two novel genes, CDC6 and ORC1, that play crucial roles in cancer development and progression. Additionally, our investigation of immune inhibitors, such as CD247, CD244, LAG3, TIGIT, and CTLA4, revealed their correlation with target genes, indicating their potential as therapeutic targets for CRC. Based on our current understanding, this is the first study worldwide to report such mechanisms in colorectal cancer, and it holds significant implications in this field. The comprehensive insights gained from this study can pave the way for finding novel innovative therapeutic agents for CRC therapy and may contribute to significant advancements in cancer therapeutics and personalized medicine.

### Supplementary Information

Supplementary Information 1.

Supplementary Information 2.

Supplementary Figure 1.

Supplementary Figure 2.

Supplementary Figure 3.

Supplementary Figure 4.

Supplementary Figure 5.

## Data Availability

The datasets generated and/or analyzed during the current study are available in the GEO datasets repository under accession **GSE18392, GSE30454**, **GSE49246, GSE59856, GSE77380, GSE38389, GSE41655, GSE70574, GSE18392, GSE30454, GSE59856, and GSE144735**.
