# Supplementary figures and images for: The integrative multi-omics approach identifies the novel competing endogenous RNA (ceRNA) network in colorectal cancer
Source: Sci Rep. 2023 Nov 9;13:19454. doi: 10.1038/s41598-023-46620-z (PMC10636147; doi:10.1038/s41598-023-46620-z)

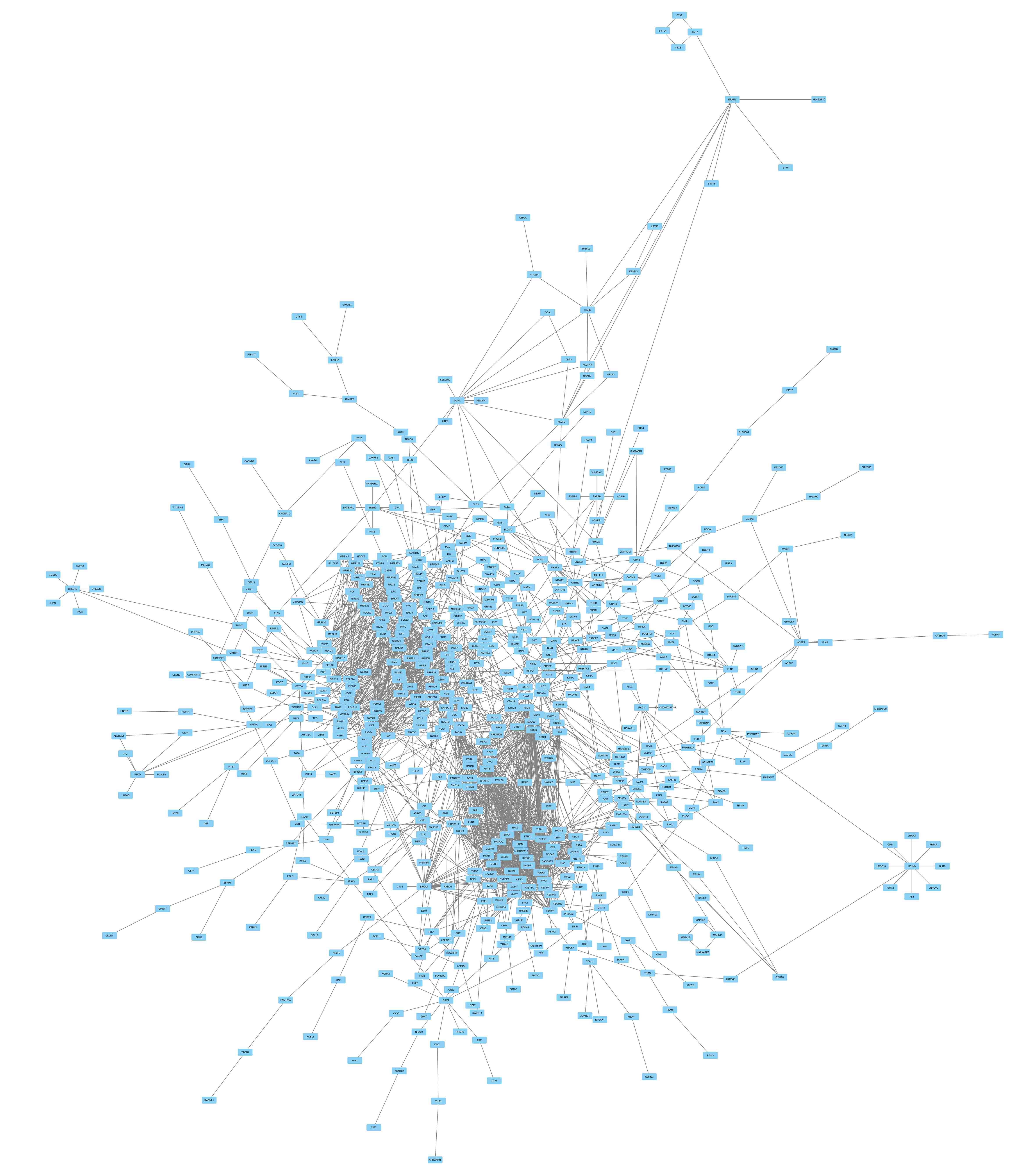

Supplement: Supplementary file 3 — Supplementary Figure 1. [file 41598_2023_46620_MOESM3_ESM.jpg]

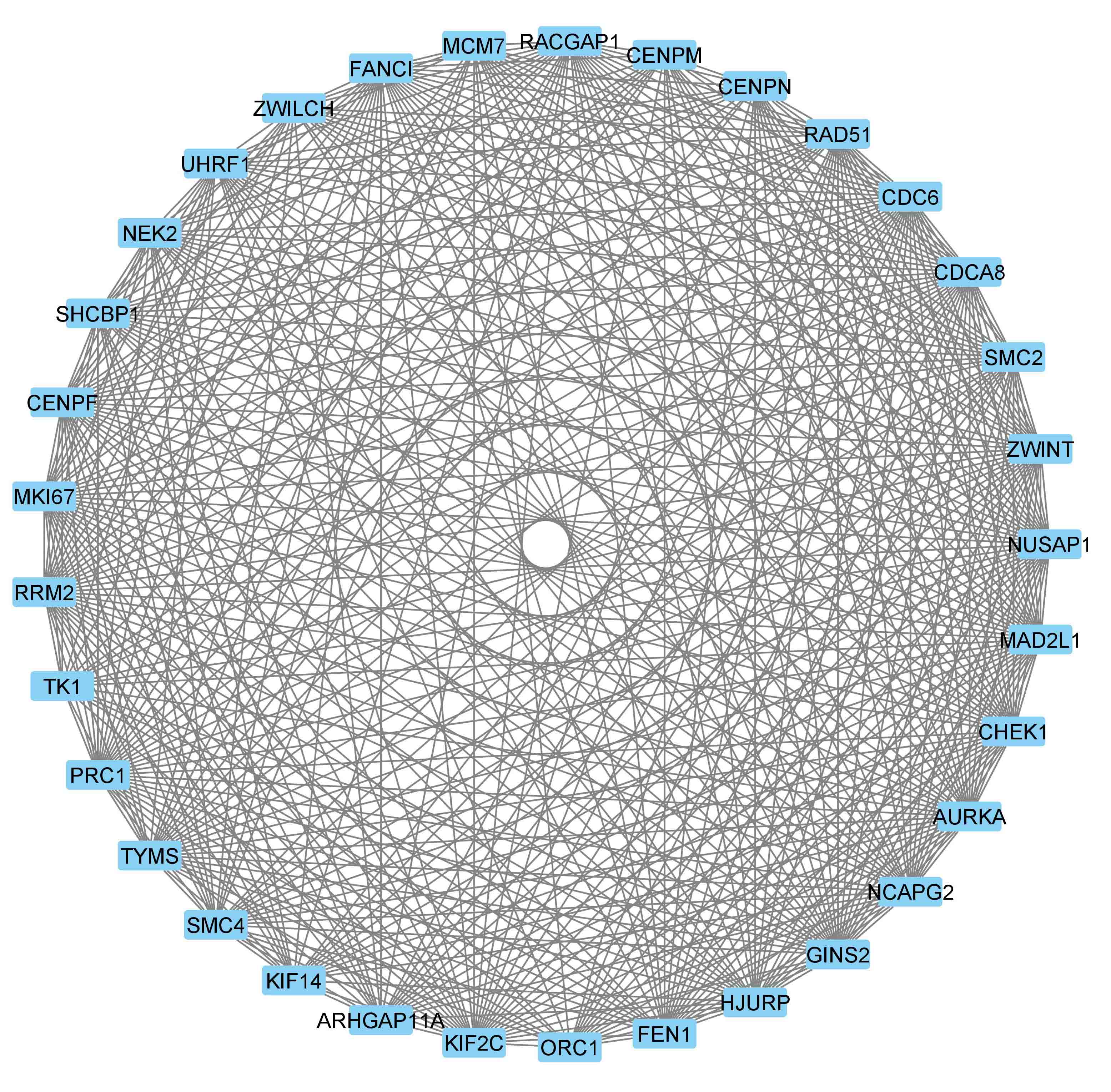

Supplement: Supplementary file 4 — Supplementary Figure 2. [file 41598_2023_46620_MOESM4_ESM.jpg]

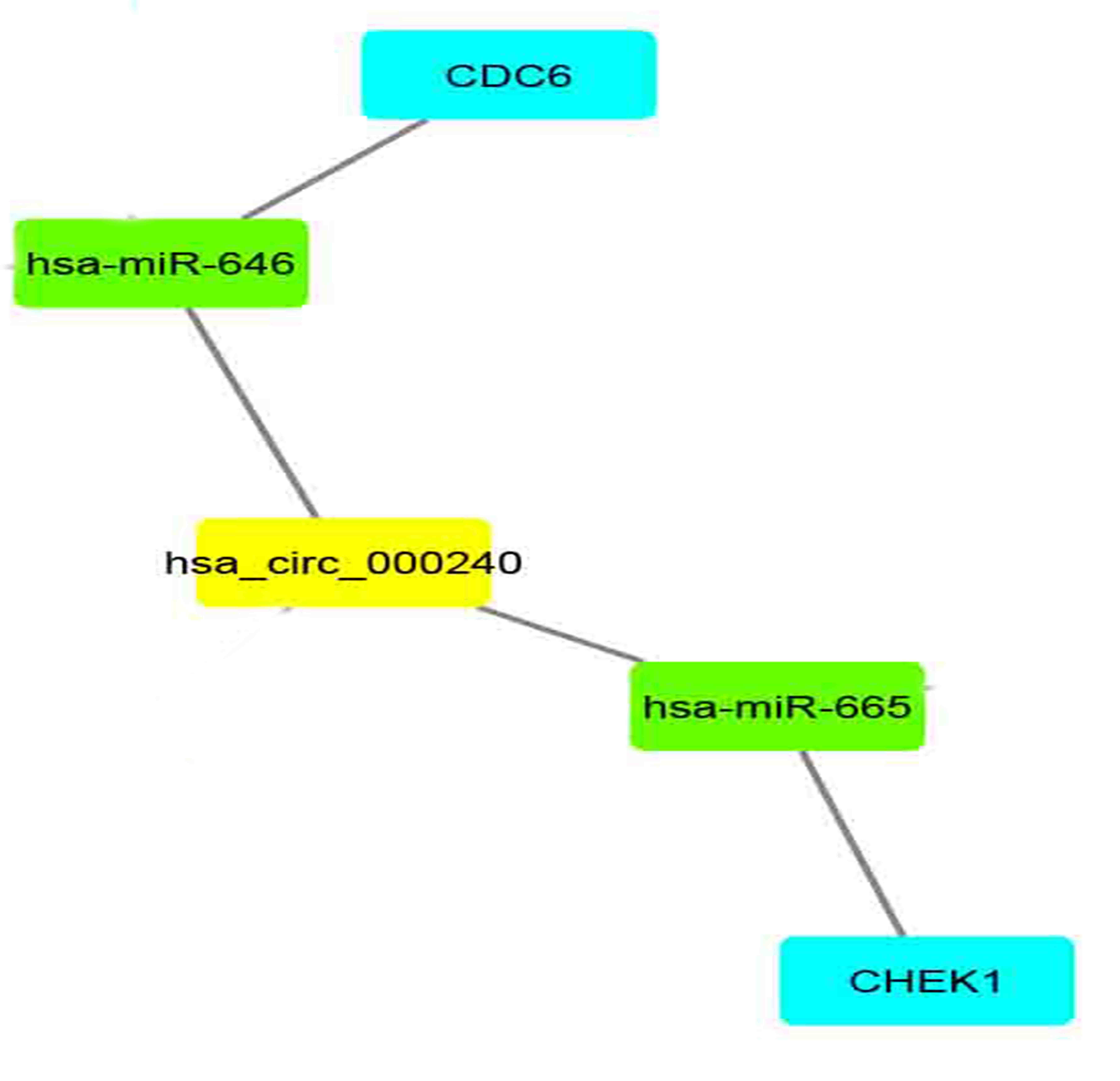

Supplement: Supplementary file 7 — Supplementary Figure 5. [file 41598_2023_46620_MOESM7_ESM.jpg]
